# Perspectives of stroke survivors, caregivers and healthcare providers on improving access to stroke care services in Tanzania: A qualitative study

**DOI:** 10.1371/journal.pone.0328334

**Published:** 2026-08-03

**Authors:** Nyagwaswa Athanas Michael, Lilian Teddy Mselle, Costansia Anselim Bureta, Yingjuan Cao

**Affiliations:** 1 School of Nursing and Rehabilitation, Shandong University, Jinan City, Shandong, Peoples’ Republic of China; 2 School of Nursing, Muhimbili University of Health and Allied Sciences, Dar es Salaam, Tanzania; 3 Department of Neurosurgery, Muhimbili Orthopedic Institute, Dar es Salaam, Tanzania; 4 Department of Nursing, Qilu Hospital of Shandong University, Jinan City, Shandong, Peoples’ Republic of China; Duke University Medical Center: Duke University Hospital, UNITED STATES OF AMERICA

## Abstract

**Background:**

Stroke is a leading cause of death and disability worldwide, with the greatest burden occurring in in low- and middle-income countries. In Tanzania, delayed hospital presentation, weak referral systems, high out-of-pocket costs, shortages of stroke-ready facilities, and limited rehabilitation services contribute to preventable deaths and long-term disability. Although previous qualitative studies have described barriers to accessing stroke care services, there remains limited evidence on strategies for improving access across the continuum of stroke care. Therefore, this study explored strategies to ensure equitable access to stroke care services in Tanzania, focusing on the perspectives of healthcare providers, stroke survivors, and family caregivers.

**Materials and methods:**

A descriptive qualitative study was conducted at Muhimbili National Hospital– Mloganzila, a national tertiary referral hospital and designated stroke center in Tanzania. A purposive sample of 45 participants was recruited, including 15 healthcare providers, 15 stroke survivors, and 15 caregivers. In-depth semi-structured interviews were conducted between June and September 2024. Interviews were transcribed verbatim and analyzed using thematic analysis guided by the WHO health system building blocks framework.

**Results:**

Thematic analysis identified six themes aligned with the WHO health system building blocks. These include: (1) raise public awareness on stroke risks, prevention and treatment; (2) strengthen stroke care resources and infrastructures at primary care facilities; (3) increase healthcare financing and stroke services insurance coverage; (4) integrate health information systems in stroke care; (5) train multidisciplinary teams in stroke care and effective communication; and (6) improve stroke care services delivery across care continuum.

**Conclusion:**

Improving access to stroke care requires both patient-centered and health system–level interventions across the continuum of care. Strengthening public awareness, healthcare infrastructures, referral pathways, service delivery, rehabilitation access, workforce capacity, and health insurance coverage may reduce inequities in accessing stroke care services in Tanzania.

## Introduction

Stroke is a leading cause of death and disability worldwide, with significant burden in World Bank low- and middle-income countries (LMICs). Globally, more than 12 million new stroke cases and over 6.5 million stroke-related deaths occur annually, with nearly 86% of deaths and 90% of stroke-related disabilities occurring in LMICs [1]. Sub-Saharan Africa (SSA) carries a disproportionately high burden due to rapid urbanization, increasing prevalence of hypertension and diabetes, and weak healthcare infrastructure. The incidence and prevalence of stroke in SSA are estimated to be 316 and 1,460 per 100,000 people respectively, while the case fatality is more than 80% [2]. In Tanzania, in-hospital mortality rate is more than 30% especially among survivors who present late to tertiary care facilities [3].

Timely access to acute stroke care and rehabilitation services is essential for reducing mortality and improving functional outcomes. Emergency treatments such as thrombolysis can significantly reduce secondary brain injury and improve recovery when delivered within 4.5 hours of symptom onset [4]. Studies show that fewer than 10% of survivors with acute ischemic stroke in SSA receive treatment within the recommended 4.5 hours window, and only 19 countries have stroke units [5]. Recent surveys of stroke services in Tanzania found that only 2 hospitals had active stroke registries, 8 hospitals had dedicated stroke units, and only 5% of survivors present at the tertiary hospitals within 4.5 hours of symptom onset [3,6]. Furthermore, more than 30% of stroke survivors do not receive formal rehabilitation services after hospital discharge leading to poor recovery outcomes [7].

Several global organizations have emphasized the need to strengthening access to acute stroke care and rehabilitation services. The World Health Organization (WHO) advocates for integrating stroke and other non-communicable diseases into Universal Health Coverage (UHC) and prioritizes stroke prevention and management in its global action plan for non-communicable diseases (NCDs) [8]. Similarly, the World Stroke Organization (WSO) and the American Heart Association/American Stroke Association (AHA/ASA) provide education materials to increase stroke awareness and early recognition, and guidelines for evidence-based acute stroke care and rehabilitative services [9]. However, translating these global recommendations into practice requires understanding the local realities such as barriers that influence access to care.

In SSA, access to stroke care services is constrained by delayed recognition of symptoms, low health literacy, weak referral systems and financial barriers. Many survivors and families fail to identify stroke warning signs, often attributing them to aging, fatigue, chronic illness, and spiritual or traditional beliefs [10]. Emergency medical services also remain underdeveloped, with limited ambulance availability, poor referral coordination, and shortages of diagnostics and experts [10,11]. Financial barriers further restrict access to stroke care services, since many survivors face substantial out-of-pocket expenses related to transport, neuroimaging, medications, and rehabilitation services. For individuals without comprehensive health insurance, these costs result in delayed treatment, poor treatment adherence and interrupted rehabilitation [12].

In Tanzania, specialized stroke services are concentrated in zonal referral hospitals, specialized national hospitals and few urban private hospitals, leaving regional and district hospitals without adequate capacity for stroke care and rehabilitation. Distance to national and zonal referral hospitals, high costs of stroke care and shortage of stroke-trained healthcare providers further contribute to inconsistent and delayed treatment across the care continuum [13]. These challenges highlight the need for context-specific strategies to improve access to stroke care services as perceived by stroke survivors, caregivers, and healthcare providers.

Although previous studies in Tanzania have identified service gaps within stroke care pathway, much of the existing literature focuses on quantitative descriptions [14,15]. Limited qualitative studies have simultaneously explored the experiences of stroke survivors, caregivers and healthcare providers in accessing stroke services across the care continuum [16,17]. However, most these studies emphasize on barriers rather than practical, context-relevant strategies for improving access to stroke care services. There is also limited application of comprehensive health-system frameworks to structure qualitative understanding of context-specific practical solutions to improve access to stroke care services. Guided by the WHO six health system building blocks framework [18], this study describes strategies for improving equitable access to stroke care services in Tanzania through the integrated perspectives of stroke survivors, caregivers and healthcare providers.

## Materials and methods

### Study design

The consolidated criteria for reporting qualitative research (COREQ) guidelines were used to ensure transparency in reporting the study methods and findings [19]. This study employed a descriptive qualitative design. A descriptive qualitative approach was appropriate because it allowed for a flexible and straightforward description of participants perceived context-specific strategies for improving access to stroke care services across the continuum of care [20]. This design ensured that findings reflect practical insights that can directly inform policy, stroke service delivery and health system strengthening in Tanzania and similar settings.

### Setting

The study was conducted at Muhimbili National Hospital (MNH)- Mloganzila, a 600 bed capacity tertiary hospital located in Dar es Salaam, Tanzania [21]. The hospital serves as one of the country’s largest stroke centers, and receives a high volume of patients, with the neurology clinic managing approximately 90 patients with stroke per day. In addition, the hospital receives referrals from district and regional referral hospitals across Tanzania, and admits about 50 stroke patients per month [21]. The hospital provides comprehensive stroke care services across the continuum of care, including emergency stroke care, ward care, critical care, as well as specialist and super-specialist stroke services. Recently, MNH- Mloganzila has been designated as the national stroke center of Tanzania, reflecting its central role within the national referral pathways.

### Study participants and recruitment

The study involved three key participant groups: healthcare providers, stroke survivors and caregivers. Healthcare providers were eligible if they were nurses or doctors with at least six months of experience in caring for stroke survivors within acute care, critical care, rehabilitation or outpatient follow-up settings. Stroke survivors and caregivers were included if they were aged 18 years or older and were able to communicate verbally. Stroke survivors were required to have a confirmed diagnosis of stroke, while caregivers had to have lived with and provided support to the stroke survivor from the onset of stroke to at least two weeks after hospital discharge. Survivors with significant cognitive impairment or altered consciousness were excluded to ensure direct participation in in-depth interviews without proxy interpretations. Stroke survivors and caregivers were recruited from the neurology outpatient clinic during routine follow-up visits, particularly at the nursing station during vital sign assessments, while healthcare providers were recruited after daily clinical meetings, ward rounds and routine service activities in their respective units or departments.

### Sampling and data saturation

Purposive sampling was used to recruit participants with diverse experiences, roles, and perspectives related to stroke care, allowing for information-rich data generation [22]. A sample size of approximately 10–20 participants per group was initially planned based on qualitative research guidance suggesting that this range is sufficient to achieve thematic saturation [23]. A total of 45 participants including 15 stroke survivors, 15 caregivers and 15 healthcare providers were recruited in the study. Equal group sizes were not predetermined as a strict requirement but were used as a practical guide to ensure a balanced representation. Saturation was monitored concurrently during data collection and analysis through regular review of interview transcripts, coding patterns and repetition of themes. Thematic saturation was achieved when no substantially new themes, insights or variations were emerging across participant groups.

### Data collection

Data collection took place from June to September 2024 using in-depth interviews (IDIs) guide informed by the WHO health system building blocks (see Supplementary File 1). Field notes and document reviews (e.g., stroke care protocols, policy documents) were also used to contextualize findings. The timing for data collection was arranged through mutual agreement and appointments with each participant. Using the IDIs facilitated a thorough exploration of participants’ experiences, opinions, and emotions [24]. The IDIs were conducted separately by the principal investigator and two trained research assistants; a female research assistant with a master’s degree in public health interviewed stroke survivors, a male research assistant with a bachelor degree in nursing interviewed caregivers, and the male principal investigator with master’s degree in nursing interviewed healthcare providers.

To ensure consistency and standardization across all interviews, all interviewers underwent prior training on the study objectives, interview guide, qualitative interviewing techniques, ethical considerations, probing strategies and reflexivity to minimize interviewer bias. Mock interviews and regular debriefing sessions were also conducted to promote consistency in questioning style, probing depth and interpretation of participant responses. The researchers allowed for a natural flow during the interviews and used probes between the main questions to capture all key information on participants’ perspectives regarding the strategies to improve stroke care services in Tanzania.

Survivors and caregivers were interviewed in a quiet room in the outpatient clinic department, while healthcare providers were interviewed in quiet rooms of neurology unit, intensive care unit and out-patient department. Only the researcher and the participant were present in the room during face-to-face interview. The interviews were conducted in Kiswahili, as both the researchers and the participants were native speakers. To reduce potential social desirability bias, participants were reassured that participation was voluntary, responses would remain confidential, no identifying information would be linked to their statements, and that the study was intended for service improvement rather than performance evaluation. Each interview was audio-recorded and lasted between 30 and 60 minutes.

### Data analysis

The study applied the Braun and Clarke’s six steps thematic analysis using initially a deductive approach [25], guided by the WHO health system building blocks (see Supplementary File 2 and 3). An inductive approach was then incorporated to allow identification of unexpected, contradictory or context-specific issues that emerged beyond the predefined WHO health system domains. This combined deductive–inductive approach ensured that the analysis remained both theoretically informed and grounded in participants’ perspectives.

The analytic process began with familiarization, where the researchers immersed themselves in the data by reading and re-reading transcripts. Next was generating initial codes, whereby an experienced qualitative researcher, trained in using Dedoose software for data analysis coded the transcripts to identify and label meaningful features across the dataset. To prevent single-coder interpretive bias, all transcripts and coding decisions were independently reviewed by another member of the research team, followed by regular inter-coder discussions to compare interpretations, resolve discrepancies, and refine the coding framework. In the third step, the codes were grouped into broader sub-themes and themes. This was followed by reviewing the sub-themes and themes, where the themes were further refined to ensure they accurately represent the data. The fifth step, involved clearly describing the essence of each theme. Finally, the themes were woven into a coherent narrative that describes the perspectives of stroke survivors, caregivers and healthcare providers on strategies to improving access to stroke care services in Tanzania.

### Human ethics and consent to participate

This study adhered to the ethical principles outlined in the Helsinki Declaration for research involving human subjects. Ethical approval was obtained from the Institutional Review Board of Muhimbili University of Health and Allied Sciences (Ref. No. MUHAS-REC-04-2024-2139). All participants provided written informed consent to participate and to have their interviews recorded, after being fully informed about the benefits and risks of participating in the study.

### Rigor and trustworthiness

The study adhered to established standards for rigor in qualitative studies including dependability, confirmability, credibility, and transferability as described by Guba [26]. To ensure credibility, multiple sources of information such as field notes and diverse study participants, and researchers with different research, academia and clinical experiences were used. Transferability was addressed by providing a rich, detailed description of the study context, participant demographics, and research setting, enabling researchers to assess the applicability of the findings to different contexts. To establish dependability, a detailed audit trail was maintained, documenting all research decisions, data collection processes, and analytical procedures, ensuring transparency and consistency in the study’s execution. Confirmability was enhanced by maintaining reflexivity, where the researcher actively acknowledged and minimized biases through journaling and monthly peer debriefing sessions. Verbatim quotations from interviews were used to illustrate the findings by accounting for participants’ perspectives of improving stroke care services in Tanzania.

### Researchers’ characteristics and reflexivity

The research team comprised qualitative and mixed-methods research experts from clinical and academic institutions. The researchers had no prior familiarity with the participants, as they were working in different academic and healthcare institutions. However, they brought a strong understanding of stroke care services delivery, informed by their research and clinical experiences.

## Results

### Demographic characteristics

Most of the stroke survivors were male who had stopped working due to functional impairment, and had experienced their first-ever stroke between 2018 and 2024 (see Table 1). While, majority of caregivers were female, aged between 28 and 73, and were caring for their close family members including father/mother, husband/wife and sister/brother. Among caregivers, six had stopped working to care for their loved ones. The healthcare providers included were physicians and nurses with an average of five years of work experience in stroke care at emergency department, stroke ward, intensive care unit, and out-patient department.

**Table 1:** Characteristics of study participants.

Variable		Healthcare Providers (n = 15)	Survivors (n = 15)	Caregivers (n = 15)
Sex	Male	4	9	4
	Female	11	6	11
Age	20-29	4	0	1
	30-39	10	0	4
	40-49	1	2	7
	50-59	0	4	0
	60-69	0	7	2
	70+	0	2	1
Marital	Single	6	0	0
status	Married	9	14	15
	Separated	0	1	0
Education level	Primary	0	8	6
	Secondary	0	4	5
	College/university	15	3	4
Residence	Dar es salaam	15	10	12
	Other cities	0	5	3

### Descriptions of the themes

**Fig 1:**
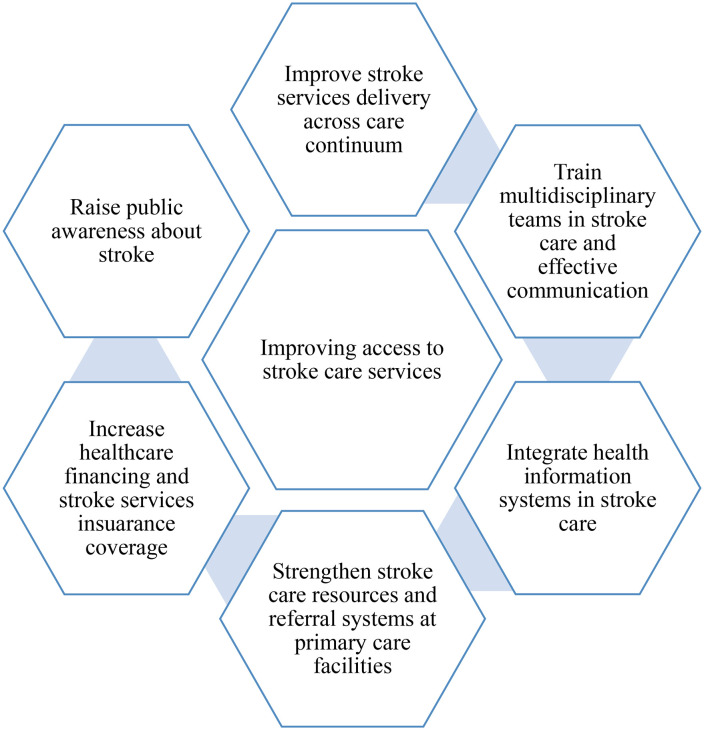
Strategies to improve access to stroke care services in Tanzania.

Thematic analysis revealed six themes that align with the WHO health system building blocks (see Fig 1). First, raising public awareness on stroke risks, treatment and prevention through mass media campaigns, social media and community drives. Second, increasing availability of stroke diagnostics, medications and experts in primary care. Third, increasing healthcare financing and stroke services insurance coverage. Fourth, integrating health information systems across healthcare facilities. Fifth, strengthen multidisciplinary teams in stroke care and effective communication. Finally, improving stroke care services delivery across care continuum.

### Raise public awareness on stroke risks, treatment and prevention

Stroke survivors and caregivers underscored the need for wider public education on stroke through radio, television and social media platforms to improve early symptoms recognition and timely healthcare seeking. They noted that radio and television remain the most accessible and trusted sources of health information, particularly for older adults and people living in rural communities, while social media platforms effectively reach younger populations and urban residents.

*Healthcare providers need to provide stroke education to the public on risk factors, prevention and treatment though radio and TV because they are available in all areas. Also, they can use social media to reach many people especially the Gen Z. Survivor#08*

*Because of the negative beliefs about stroke in the community, healthcare providers should develop social media, radio or TV programs to educate the public about the causes and treatment of stroke. Caregiver#07*

However, healthcare providers also emphasized the need for tailoring and delivering right information about stroke from local people such as stroke peers, church leaders and community respected individuals. They believed that right information from local people in the community would help to increase trust and acceptance of the messages, and reduce misinformation, challenge superstition beliefs and promote better health seeking behaviors.

*Information from church leaders can help people to find hope when they are struggling, and it can really reduce stress and treatment delays especially during the first days of stroke onset and hospitalizations. Healthcare provider#08*

Participants also identified the need for healthcare providers-led community-based outreach programs to spread stroke education, and health checks for blood pressure, sugar and cholesterol. They perceived that scaling-up cost-effective screening measures at the communities would increase mass awareness for stroke and other non-communicable diseases.

*I think healthcare providers should come to our communities and give us education about stroke. This can also help us to get aware about stroke and do checkup on time. Caregiver#14*

*Increase education and screening for non-communicable diseases. Encourage people in the streets to do regular checkups for blood sugar, pressure and fat. Healthcare provider#09*

Interestingly, some healthcare providers emphasized the need for a proactive and long-term approach to integrate stroke prevention education into the primary school curriculum. They believed that introducing this knowledge early in life would help to build a foundation of awareness about stroke, thereby creating a ripple effect of stroke awareness in the community.

*I think health education for non-communicable diseases like stroke should be provided at schools. Children should be taught to know the normal blood pressure and sugar because they can pass this information to their parents. Healthcare provider# 05*

### Strengthen stroke care resources and referral systems at primary care facilities

Participants emphasized the importance of increasing the availability of stroke diagnostic services at primary care health facilities. They explained that improving access to imaging services such as CT scans and MRI at lower-level facilities would allow faster decision-making thereby increasing chances of receiving timely treatment and referral.

*The government should prioritize stroke care and ensure availability of brain CT and MRI at primary care facilities. Availability of diagnostics will ensure that people are diagnosed, treated, and recover early so that they continue with their activities. Caregiver#01*

*There should be improvement in stroke services such as availability of CT and MRI in primary care facilities to ensure timely investigations, and referral to tertiary hospitals. Survivor#07*

Participants also emphasized the importance of ensuring access to antihypertensive drugs, antiplatelet therapy, diabetes medications, and emergency drugs at primary healthcare facilities. They believed that reliable medicine supply would strengthen acute, prevention and long-term management of stroke while reducing financial burden on accessing stroke care services.

*I think they need to improve stroke care services in our regional and zonal referral hospitals. In particular, the government should ensure availability of stroke medications and other services near our homes. Stroke survivor#05*

*My suggestion is that the regional and district hospitals need to be equipped with all medications for stroke, rather than having the patient go from district hospitals to tertiary hospitals for acute management or medication refill, which is expensive. Caregiver#05*

While healthcare providers stressed on the importance of improving stroke knowledge among healthcare providers working in primary care settings. They viewed task-sharing and continuous professional development of nurses and general physicians as practical approaches for improving stroke care quality in primary care facilities where neurologists and medical specialists are scarce.

*Another thing that we need is to improve our primary healthcare system staffing level and capacity. We need to train nurses and general physicians to provide better stroke care. Healthcare provider#10*

Other healthcare providers suggested that hospitals should establish stroke-triage systems to ensure that stroke survivors receive timely initial treatment and referral. While, caregivers suggested that well-equipped and staffed ambulances should be used to transport stroke survivors from primary care facilities to stroke-capable hospitals.

*Most of patients don’t arrive on time, even if it’s to stabilize them you can’t because time has passed, (…). So, the primary care facilities should have better triage and referral systems to avoid delay. Healthcare provider#11*

*I suggest that if they refer a patient, they should first stabilize the patient and handle everything, not just halfway, as it is not healthy. A nurse should also be there during referral to handle any emergency that can happen on the way to the hospital. Caregiver#09*

### Increase healthcare financing and stroke services insurance coverage

Participants believed that increased budget allocation for health sector can reduce out-of-pocket payments for essential stroke services. They suggested that subsidizing key stroke services such as CT scans and MRI, rehabilitation services and essential medicines would improve affordability and accessibility of stroke care services across all levels of healthcare facilities.

*The government should facilitate the process and procedures for buying medical equipment and medications so that they are available at a reasonable low cost. Stroke survivor#01*

*But as I said, majority are unable to afford the cost of care because it is very expensive. So, government efforts to subside costs of medications, CT scan and MRI are important to ensure stroke care services are accessible and affordable. Healthcare provider#02*

Participants also mentioned the need for special schemes for elderly group and other people who can’t afford healthcare insurances. Though, many participants acknowledged that health insurance schemes play an important role in protecting families from catastrophic health expenditures.

*If the government could consider treatments for the elderly people or those who are less fortunate, it would help because we want them to get treated, but they don’t have health insurances to cover hospital bills. Caregiver#06*

*With the healthcare insurance, I receive services without paying at hand. This helps me to come to the hospital regularly, because I don’t feel so much financial burden when I go to the hospital. Survivor#04*

However, stroke survivors and caregivers stressed that wider insurance coverage would encourage earlier healthcare seeking, improve adherence to treatment and reduce treatment interruption. They suggested that healthcare insurances should include aspects of stroke care such as routine stroke screening, medications refill and rehabilitation services.

*I’m thankful that my health insurance is accepted here at the out-patient department, but it doesn’t cover routine screening and rehabilitation services. Therefore, the government should ensure that the health insurances cover all healthcare services. Stroke survivor#06*

*Sometimes you’re prescribed medications, but you are told to go to find them elsewhere because the insurance doesn’t cover. So, I suggest the insurances should be comprehensive enough to cover all aspects of stroke care. Caregiver#06*

### Integrate health information systems in stroke care

Participants emphasized the need to strengthening health information systems to improve continuity and quality of stroke care across different levels of the healthcare system. They believed that linking health information systems would support faster clinical decisions, reduce unnecessary delays and improve stroke care access across the care continuum.

*There should be integrated electronic system that allows to transmit data between hospitals so as patients can be seen by primary care physicians during follow-up clinic. Healthcare provider#13*

*But importantly is to link information systems across hospitals so that personal information can be retrieved across hospitals by using health insurance or national identity card to prevent delays in accessing care and repetition of investigations. Caregiver#13*

Participants also painted the use of SMS reminders and phone calls as simple and practical strategies to improve access to stroke care services. Regular SMS and phone communication were thought to remind stroke survivors about clinic appointments, medication schedules and rehabilitation sessions, and provide an opportunity to seek clarifications without the immediate need to travel long distances to the hospital.

*Our communication department can develop stroke call center and an SMS reminder system for the next of kin or stroke survivors so they know their clinic appointments and we know their progress while at home. Healthcare provider#14*

*Healthcare providers can use SMS reminders and phone calls to guide us on diet, and remind us on important things such as medication schedules, stroke warning signs, and home-based exercises and clinic dates. Caregiver#02*

Furthermore, participants consistently expressed the urgent need to digitalizing health information systems as a key enabler for improving stroke care access. Participants believed that digitizing stroke care services and improving real-time virtual communication could ensure timely access to care and enhance care continuity after hospitalization.

*We should utilize telemedicine so that we can have virtual conversations with stroke survivors and their families because some of them delay or fail to come at the hospital due to financial issues. Healthcare provider#11*

*If other means for follow-up care such as phone calls or virtual platforms are deployed then I can communicate with the doctors while at home. Survivor#05*

### Train multidisciplinary teams in stroke care and effective communication

Training multidisciplinary teams (MDT) in stroke care pathway covering aspects of rapid assessment, stroke protocols, emergency interventions, complication prevention, stroke rehabilitation best-practices, and multidisciplinary care approach emerged as critical concepts to ensure provision of high-quality stroke care services at tertiary hospitals.

*All frontline healthcare providers should receive on-job training about stroke rapid assessment, stroke protocols, emergency interventions, complication prevention, stroke rehabilitation best-practices, and multidisciplinary care. Healthcare provider#14*

Participants also underscored the need for strengthening multidisciplinary team collaboration and communication to improve the quality of stroke care services at tertiary hospitals. They explained that when healthcare providers are trained, and work together with shared knowledge and clear roles, stroke care moves beyond in-hospital treatment to comprehensive long-term care.

*Another thing, there should be a multidisciplinary stroke team. Good collaboration among the healthcare providers can help to improve quality of stroke care services across care continuum. Healthcare provider#05*

*(…), I spent some hours without receiving stroke medicines in the ward, (…). So, improving communication and collaboration among healthcare providers can prevent this scenario to happen to other patients. Survivor#10*

However, caregivers expressed frustration when they received inconsistent care information from healthcare providers. Some preferred direct doctor-led communication because nurse-delivered information sometimes felt incomplete. Nevertheless, healthcare providers suggested that nurses are better positioned to communicate and coordinate stroke care within the multidisciplinary team.

*I think it would be better to have direct communication with doctors, because when you get it directly from the doctors, the doctors say exactly what they saw. But with the nurses, they might leave out some key information. Caregiver#09*

*Nurses should be trained to know stroke and its treatment so they can educate patients and their relatives about the disease and its treatment plans. Also, when nurses are trained, they can coordinate stroke care within the multidisciplinary team. Healthcare provider#02*

Importantly, participants highlighted the need for increasing peer-led and informal caregivers’ training to enhance early community reintegration after stroke. Participants mentioned that peer-led stroke clubs could add a supportive layer by providing emotional support, rehabilitation guidance, lifestyle modifications and medication adherence strategies. While training informal caregivers was thought to enhance caregivers’ ability to care with love, courage and competence, thereby assisting the stroke survivor to navigate smoothly through the recovery journey.

*We need social meetings or clubs like those which are done by occupational therapists for stroke rehabilitation. These clubs are important because they bring people together as a community during recovery. Healthcare provider#09*

*Well, if they had educated us on what kind of care a patient with this condition should receive, like specific practices or exercises, it would have been helpful for us to be informed to take good care of our patient and keep-up with clinic appointments. Caregiver#12*

### Improve stroke care services delivery across care continuum

Participants recognized that improving stroke care service delivery is critical for enhancing patient outcomes, reducing preventable disability, and ensuring equitable access to stroke services. Healthcare providers suggested that tertiary hospitals should improve care at the acute stroke units, by adopting stroke protocols and time targets such as “door-to-needle” and “door-to-groin” to guide provision of timely interventions and standardized stroke treatments across care facilities.

*(…), timely arrival and admission to acute stroke unit will ensure that patients undergo a CT scan, and get treatment within 4.5 hours after stroke resulting in better clinical outcomes and quality of life. Healthcare provider#11*

*But now we don’t have stroke care protocols, we do as usual, or as a routine. But I think the stroke care checklists and protocols are important to ensure we deliver high-quality stroke care. Healthcare provider#05*

Stroke survivors and caregivers expressed the need to modify the discharge care practices so that they are involved and prepared for home care. They recommended that discharge planning should be done in advance so that caregivers are taught about basic care practices, safety considerations and prevention of complications at home. They also suggested provision of stroke educational materials during discharge to increase their knowledge and skills in homecare after discharge.

*We find it difficult to handle care after discharge. (…), if we are adequately involved in care processes from admission to discharge, it could help us to take good care of ourselves at home. Survivor#12*

*Also, apart from involving us in care processes and decisions, printed materials would help us to increase knowledge of caring stroke patients, because we can refer to them while at home. Caregiver#15*

Participants also emphasized the importance of strengthening follow-up care as a key strategy for improving care continuity and long-term rehabilitation outcomes among stroke survivors. They explained that clear follow-up plans and periodic home visits led by community health workers or nurses will allow healthcare providers to monitor recovery progress, provide nursing care, administer rehabilitation exercises, and reinforce medication adherence and lifestyle adjustments.

*We don’t follow-up patients at home because of shortage of staff. If nurses could follow them at home, it can help to reduce post-discharge complication such as aspiration pneumonia and bedsores. Healthcare provider#01*

*Ah, (…), once you leave the hospital, you just have to do what they’ve instructed you to do, which is difficult for short time to master. But I suggest that nurses should visit us at home, and teach us more. Caregiver#03*

*One cannot understand what he was taught in the hospital just at once.. Continuous education and follow-up at home can increase our understanding of the importance of clinic visits and home exercises. Survivor#15*

## Discussion

The current study identified practical and context-specific strategies for improving access to stroke care services in Tanzania by integrating perspectives from stroke survivors, caregivers, and healthcare providers. The findings highlight six interrelated priorities: raising public awareness, strengthening primary care capacity, improving healthcare financing and insurance coverage, integrating health information systems, enhancing multidisciplinary team capacity, and improving service delivery across the stroke care continuum. These findings reflect the everyday challenges faced by stroke survivors, caregivers, and healthcare providers within the stroke care continuum as consistently reported in previous studies [15,16]. However, this study extends existing knowledge by identifying actionable solutions to improve access to stroke care aligning with the Tanzanian health policy and WHO priorities for preventing NCDs.

Raising public awareness on stroke risks, prevention, and treatment emerged as a critical strategy for improving timely care-seeking behavior. Consistent with prior studies in SSA, poor recognition of stroke symptoms and misconceptions about causes of stroke contribute significantly to delays in hospital presentation and missed opportunities for acute interventions [10]. Participants in this study emphasized the importance of using widely accessible platforms such as radio, television, and social media to deliver continuous, culturally relevant health education. This is particularly relevant in Tanzania where radio and television remain the most accessible sources of health information. Integrating stroke education and community drives into existing hypertension and diabetes screening programs within the Tanzanian primary healthcare system could also strengthen prevention efforts, consistent with the national NCDs policy and WHO recommendations for integrated chronic disease management [8,27].

Nevertheless, increasing healthcare insurance coverage and financing mechanisms was believed to tackle financial barriers to accessing stroke care services in Tanzania. Currently the National Health Insurance Fund (NHIF) and Improved Community Health Fund (iCHF) provide some financial protection, but coverage for stroke care services remains limited. Previous studies have shown that out-of-pocket payments delay treatment decisions, increase access gap and contribute to household poverty [12,28]. Improving financial protection for stroke-related services including imaging, medications, and rehabilitation services is important for reducing inequities in accessing care and preventing catastrophic healthcare expenditures. These findings support ongoing policy discussions around universal health coverage and highlight the need to prioritize stroke services within national health financing frameworks to ensure equity, improve health and protect families from overwhelming out-of-pocket payments [29].

Strengthening stroke care resources and infrastructure at primary care level was another key thematic area for improvement. Patients first present to lower-level facilities, yet these settings lack basic diagnostic tools, trained personnel, and clear referral systems. Previous Tanzanian studies reported that limited capacity at district and regional hospitals contributes to delayed diagnosis, inconsistent treatment and overcrowding at tertiary centers [3,30]. Rather than relying solely on expanding specialized stroke units, participants in our study recommended equipping primary care facilities with essential tools and capacity for early recognition, stabilization, and referral. Strengthening capacity of frontline healthcare workers’ at primary care facilities was also deemed to represent a more feasible and scalable strategy compared to developing resource-intensive stroke care models like those implemented in high-income countries [31]. These approaches reflect broader health system strengthening efforts to decentralize healthcare services and integrate NCDs care into primary healthcare.

The integration of health information systems was also identified as an important strategy for improving access to stroke care. Gaps in health management information system such as incomplete records, poor information exchange between facilities, and weak interoperability can constrain clinical decision-making and health system performance [32]. A recent study in Tanzania has identified that the adoption of health information system has resulted in measurable improvements in real-time data availability, patient record accuracy, and clinical workflow efficiency [33]. In the context of stroke care, where timely decision making and continuity of care are essential, integrated systems such as tele-strokes can significantly improve access to stroke care through virtual consultations. However, barriers such as unstable internet connectivity, unreliable electricity supply, limited digital literacy, and cost of maintaining digital platforms are likely to affect their adoption [33,34]. As such, strengthening phone call communication and standardized paper-based systems alongside gradual digital integration may offer a more realistic pathway before full tele-stroke systems are implemented in Tanzania and similar settings.

Furthermore, training MDT and improving stroke care communication emerged as essential element for delivering coordinated and patient-centered stroke care. Participants emphasized that effective stroke management requires collaboration and inputs from healthcare providers, family caregivers and stroke peers. This aligns with evidence highlighting that organized in-hospital and community-based MDT-led care can improve clinical care pathway, functional status and quality of life outcomes of survivors [35,36]. Notably, participants highlighted tensions in care communication, with caregivers expressing a preference for doctor-led explanations while recognizing the central role of nurses in patient education. This underscores the need for consistent team-based communication strategies to improve trust, collaboration, treatment adherence, and patient satisfaction.

Moreover, improving stroke care service delivery across the continuum was identified as a unifying priority linking all themes. Standardizing clinical protocols, improving discharge planning, and ensuring continuity through follow-up and community-based care were highlighted as key strategies. A previous study in Tanzania has shown gaps in coordination from acute management to follow-up care that lead to delayed interventions and poor clinical outcomes [37]. Thus, there is a need to move beyond hospital-centered models toward more integrated systems that include home-based care. Evidence shows that when untrained family caregivers assume the caregiving responsibilities after discharge can lead to inconsistencies in care delivery and increased risk of adverse events at home [38]. On contrary, other studies have shown that trained family caregivers have a potential to enhance treatment adherence and improve recovery outcomes [39,40]. Subsequently, locally developed nurse-trained family caregiver models warrant further evaluation as a potentially feasible and cost-effective approach to post-stroke care in Tanzania and similar settings, with possible benefits of enhancing community-based care while reducing congestion at tertiary hospitals.

### Recommendations for policies, practice and research

Governments should increase research funding for stroke treatment innovations and rehabilitation methods, ensuring that healthcare providers have access to the latest medical advancements. Furthermore, stroke care guidelines should be developed and standardized to ensure that all stroke-ready hospitals follow best practices for stroke diagnosis and treatment. Moreover, research should be done to evaluate the feasibility of stroke telemedicine and nurse-led transitional care models to ensure stroke care continuity within the Tanzanian healthcare system.

### Study limitations

This study has several limitations. First, it was conducted in a tertiary hospital, which may not fully represent the needs, perspectives and experiences of healthcare providers working in primary healthcare settings, as well as stroke survivors and caregivers who face substantial barriers to reaching tertiary care facilities. Second, there may be recall bias among stroke survivors and their caregivers when asked to recall past experiences related to accessing care at primary care facilities, as their memories could be influenced by emotional factors or time. Third, other important multidisciplinary cadres such as physiotherapists, occupational therapists, speech therapists, radiology staff, emergency medicine personnel, nutritionists and social workers were not included because of limited availability during the study period. To address these issues, the study included survivors and caregivers from both urban and rural areas, as well as those referred from primary healthcare facilities, to ensure a broader and more representative sample. To reduce recall bias, the study used a triangulation approach, combining semi-structured interviews with field notes to validate the responses of participants.

## Conclusion

Addressing challenges in stroke care in Tanzania requires context-specific strategies that respond to the realities experienced by stroke survivors, caregivers and healthcare providers. There is a need to raise public awareness about stroke, improve referral systems, and expand availability of neuroimaging, essential medicines and rehabilitation services at primary healthcare facilities. Strengthening multidisciplinary stroke teams, integrating health information systems, improving acute and post-acute stroke care, and ensuring financial protection through universal health coverage are other practical top priorities to improve access to stroke care in Tanzania.

## Supporting information

S1 Data Minimal Data Set. (DOCX)
