# Supplementary material for: Perspectives of stroke survivors, caregivers and healthcare providers on improving access to stroke care services in Tanzania: A qualitative study
Source: PLoS One. 2026 Aug 3;21(8):e0328334. doi: 10.1371/journal.pone.0328334 (PMC13432098; doi:10.1371/journal.pone.0328334)
Supplement: S1 Data — (DOCX) [file pone.0328334.s001.docx]

Minimal Data Set

| Media Title | Excerpts | Codes Applied |
| --- | --- | --- |
| INTERVIEW 4 ENGLISH VERSION.docx | Financially, there have been challenges as well, because I have to cover all her needs, including food. We were supposed to come to the clinic, but due to these challenges, we've only been able to come today. | financial struggle after stroke, skipping appointment |
| INTERVIEW 4 ENGLISH VERSION.docx | Honestly, they helped me very well, really well. I didn't expect them to assist me in that way. They were with me every step of the way, shoulder to shoulder, until the last day when we were discharged. They even helped to get her out of bed and into a chair, which was very good. Even when I went home, everything was fine. Whenever I noticed she wasn't doing well, I had a phone number to call for advice. SMS reminders and phone calls follow-up are really important for ongoing care after stroke. Healthcare providers can use this method to guide us on what foods to give patients, and remind us on important things line medication schedules, physiotherapy and clinic appointments. | support from healthcare providers, information on food at home, good services, good communication with healthcare providers, freedom to contact healthcare providers, diet instructions after discharge |
| INTERVIEW 4 ENGLISH VERSION.docx | In terms of cooperation, they would inform me if there was a specific medication that needed to be purchased because it would be better for the patient. I would buy the medication and give it to my mother, and her condition would improve. | family involvement, cooperation with healthcare providers |
| INTERVIEW 4 ENGLISH VERSION.docx | Whenever I came to check on her, they would inform me about her condition. For instance, they would tell me that her blood sugar was around twenty yesterday, and today, this morning, it was fourteen. So, I could see the progress myself. | family involvement, good communication with healthcare providers, told about blood sugar |
| INTERVIEW 4 ENGLISH VERSION.docx | I was doing the exercises at home; I never received any at the hospital. | exercise at home |
| INTERVIEW 4 ENGLISH VERSION.docx | When I took her home, I used my own understanding and intuition. No, I wasn't instructed that she should do specific exercises. I made sure she was lying down properly and then stretched her limbs, holding her firmly to help her stand and walk. I would do this every day, ensuring she walked around at least ten times. I did this daily to help her sit and move better. | exercise at home, exercises without training |
| INTERVIEW 4 ENGLISH VERSION.docx | but suddenly, she couldn't speak or do anything. So, we went straight to RRH, where they did a CT scan and discovered she had a stroke. We were then sent to tertiary hospital. After staying for couple of days at the hospital, we were discharged home. | referred to big hospital |
| INTERVIEW 4 ENGLISH VERSION.docx | So, we went straight to Regional Hospital, where they did a CT scan and discovered she had a stroke. | stroke investigations done |
| INTERVIEW 4 ENGLISH VERSION.docx | We were then sent to tertiary hospital. After staying at tertiary hospital, we were discharged home. |  |
| INTERVIEW 4 ENGLISH VERSION.docx | They then referred us here, and since we arrived, she's been getting better, and we thank God. Her eyes had even started to turn, which was a bad sign, and the staff did their best. We rushed her here, and we're grateful that she's improving now. | referred to big hospital, support from healthcare providers, religious comfort |
| INTERVIEW 4 ENGLISH VERSION.docx | I was involved in care related to managing her blood sugar. They told me which medications to buy, the ones prescribed to me, and there were some vitamins to replenish her body | family involvement |
| INTERVIEW 4 ENGLISH VERSION.docx | Yes, I don't know. But I remember when we went to the last clinic, they mentioned something about exercises, but I wasn't given specific instructions, so I just went home. | information about exercise |
| INTERVIEW 4 ENGLISH VERSION.docx | Yes, I didn't understand because they just mentioned exercises. I didn't find anyone to guide me, so I just went home. I know basic exercises like stretching, but I don't know specific ones for stroke | information about exercise |
| INTERVIEW 4 ENGLISH VERSION.docx | They told me that your patient might be discharged within these two days. They told me this, but they didn't mention anything about payment, though I knew I would need to pay. | discharge notice |
| INTERVIEW 4 ENGLISH VERSION.docx | I was only given instructions on how to administer the medications and then I left | medication instructions after discharge |
| INTERVIEW 4 ENGLISH VERSION.docx | people involved included church members, the pastor, and my family, including my siblings. We had prayers and services at home where people came to participate in prayers and worship. | family support, religious comfort, prayers at home |
| INTERVIEW 4 ENGLISH VERSION.docx | Support included the pastor who came for prayers and provided guidance. He had faced a similar issue, so he advised us on what should be done, including exercises and dietary restrictions. He also provided financial support when he could. | Involving church leaders in stroke education |
| INTERVIEW 4 ENGLISH VERSION.docx | We are a family with limited resources. My mother had been managing her business on her own. After she encountered this issue, managing the situation became challenging due to limited resources, including financial constraints which prevent us to come for hospital visits. | financial struggle after stroke, skipping appointment |
| INTERVIEW 4 ENGLISH VERSION.docx | Challenges in the hospital included the need to purchase specific medications which I sometimes couldn't afford at that moment. This was a significant difficulty, as the required medications were essential for my mother's treatment | cost of medications |
| INTERVIEW 4 ENGLISH VERSION.docx | For example, you might be told to buy a certain medication, but at that time, you don't have the means. That was a challenge for me. Sometimes you are told that a certain medication is needed urgently to help the patient, but you might not have the resources to purchase it at that moment. | financial struggle after stroke, cost of medications |
| INTERVIEW 4 ENGLISH VERSION.docx | I mean, I don't know her current condition because we haven't done a scan. Yes, since the last time, I don't know exactly how she is, but I've just been given medication to use. | inadequate information, improve communication |
| INTERVIEW 4 ENGLISH VERSION.docx | When I was leaving, I wished they had told me something like, "Your mother is fully recovered." I don't know how to explain it. | inadequate information, improve communication |
| INTERVIEW 4 ENGLISH VERSION.docx | I wasn't given any education, but I was just told to see the diabetes doctor, which I didn't do that day. So, I was scheduled for a return visit, and when I went back, they provided education on how to care for her diabetes. So they need to provide also education about stroke at the clinic, or through radio and TV, and recently social media like Instagram, Facebook, WhatsApp and YouTube | Stroke education on radio and TV, stroke education on social media |
| INTERVIEW 1 analysis.docx | However, it was my first time seeing a stroke patient, so as a human being, it was something new, I was scared and didn't understand where it would end because it was my first experience | stroke is scary for first timers, need stroke education |
| INTERVIEW 1 analysis.docx | You need to be patient and support the patient because you are the person closest to them and the only one who can provide support. The nurses and doctors told me that I was the only person close to him, so I was the one who could support and help him, and more importantly, encourage him regarding the disease because it's not something that heals in one day; it takes a long time. He had a stroke on the right side, which also caused speech problems. | Good commination builds trust and confidence |
| INTERVIEW 1 analysis.docx | So, I would say that normally, when a disease like this enters a family, a person becomes confused and doesn't understand, and that's how it was for us | stroke worries the family, need for stroke education |
| INTERVIEW 1 analysis.docx | Everyone tries to give alternative advice: let's go here to specialists (traditional healers), but I think that sticking to the hospital issue is the right place because every time I went to the hospital and saw the doctors, I saw that there was progress we were making, even if you can't see it with your eyes, but I, who stays with him, know that we have made this step. Because of the negative beliefs about stroke in the community, people should be educated about stroke so that they understand its causes and treatment. Healthcare providers can develop radio or TV programs to educate the public. Also, social media have been widely used by gen Z, so it is another way to reach and deliver message to them | need for stroke education |
| INTERVIEW 1 analysis.docx | You have to encourage them to follow the instructions given by the doctor, especially exercises, to stand again and continue with their family | encourage survivors to follow instructions, role of caregivers |
| INTERVIEW 1 analysis.docx | So, maybe I should say that you need to have patience because there are times when they were doing everything, but now they can't, and they might tell you in a harsh tone because even if it were you, you would feel the same way. So, you just need to be patient and listen to what they are saying, but later they will see that what they are saying may not be right. | role of caregivers |
| INTERVIEW 1 analysis.docx | Don't leave them at home; take them to the activities they were doing every day, even if they can't do them, but seeing will show them the importance of struggling to return to their position. | role of caregivers |
| INTERVIEW 1 analysis.docx | Something I insist on, which I have heard from many people, is that when someone gets a stroke, many people start thinking about alternative treatments, maybe from traditional healers and such things. But remember, someone who gets a stroke needs to exercise every day. When you take them to a healer, there is no exercise equipment; even the diet, they can't meet with anyone, they are locked up somewhere. So, I am sure you are going to destroy them. | need for stroke education |
| INTERVIEW 1 analysis.docx | When I was in the hospital continuing with exercises, the doctor told me that this person was lost for a year and he went for those alternative treatments, but when they returned, they came back worse because there were no exercises there, nothing was happening | need for stroke education |
| INTERVIEW 1 analysis.docx | if you get such a challenge, don't take it as a very difficult challenge because I believe in God, and God can tell you to do something, and He will do His part. So, looking at other treatments, in my opinion, is not right because I stuck to the whole issue of hospital treatment, following experts' instructions and schedules, and until now, as I am speaking to you, my relative is fine | religious comfort in stroke care |
| INTERVIEW 1 analysis.docx | So, it's not a difficult thing if you decide because if you decide, now they say I don't want to see him as he is, I want to see him as he was before. You will do it, but if you lose hope and see it as a burden, you won't take him to exercise, and he will always be a burden because he will continue to be sick | dedication in caregiving |
| INTERVIEW 1 analysis.docx | The challenge was significant because it also required a lot of my time to invest in that initial period. Remember that during that time, he couldn't do anything, so I had to be close at all times | stroke is time consuming |
| INTERVIEW 1 analysis.docx | They also gave me the freedom to contact them anytime I faced difficulties. So, I was at home but had a good opportunity to communicate with them whenever I encountered challenges | freedom to contact healthcare providers |
| INTERVIEW 1 analysis.docx | Even when I returned to the hospital for clinic visits or exercises, the way they received me was still encouraging, showing that this journey is possible. | support from healthcare providers |
| INTERVIEW 1 analysis.docx | So, I can say one thing: the journey of a patient like this, nurses play a very big part because if a nurse doesn't treat you well, even you, because you don't know the condition, you will lose hope. A nurse is the first person who tells you that this disease exists and people recover. After getting the information, they guide you on what to do. So, I received a lot of help from the nurses. | support from nurses |
| INTERVIEW 1 analysis.docx | they provided guidance on all services. They showed me the exercises we were doing in the morning, instructing me to repeat those exercises in the evening at home, ensuring the patient reaches certain milestones. So, they involved me in all the important steps. | family involvement, instructed on home exercises |
| INTERVIEW 1 analysis.docx | But also, when I reached the ward, I didn’t have any bad event; only good ones because I was receiving good services. I didn’t encounter any bad events | services received in the ward |
| INTERVIEW 1 analysis.docx | There are many patients with similar conditions, and I think such patients need a certain amount of time because when I started at another hospital, you could see clearly if a doctor was attending to the patient, but also there were many other patients | stroke affect many people |
| INTERVIEW 1 analysis.docx | So, they need more staff, and also, machines because there are many patients with such problems. You find that the machine is in high demand. When the patient finishes hand exercises and wants to use the machine, they wait for a long time and get tired. Because of the nature of the illness, the patient gets tired. Another one might say, "Take me home, we’ll do it tomorrow." But you need to do it. | staffing and machines at physiotherapy, congestion at tertiary hospital |
| INTERVIEW 1 analysis.docx | Home becomes another hospital. Once they leave the hospital, they need to be handled as required, but when they return home, it’s like starting another hospital all over again | home care should reflect hospital care |
| INTERVIEW 1 analysis.docx | love must be present to ensure you follow the doctor's instructions and other things. | care with love |
| INTERVIEW 2 ENGLISH VERSION.docx | We started with tests and treatments until today when we came here, we have been referred by another hospital, saying that this place has many neurologists. | referred to big hospital |
| INTERVIEW 2 ENGLISH VERSION.docx | First, he was the breadwinner, as is common in our African families, and when he was bedridden, I had to take on all responsibilities, nursing him and ensuring the family continues | financial struggle after stroke |
| INTERVIEW 2 ENGLISH VERSION.docx | Eventually, with advice from others and doctors emphasizing the importance of exercise, he started doing physiotherapy here at the hospital | support from healthcare providers |
| INTERVIEW 2 ENGLISH VERSION.docx | This queue problem, I think, is a chronic issue, or maybe we have too many patients for the space. We really crowd, honestly. Even health-wise, I think it's not just us but also the healthcare workers who are at risk because we are packed in so tightly. You might report here in the morning but find yourself leaving in the evening. | crowd at tertiary hospital |
| INTERVIEW 2 ENGLISH VERSION.docx | The way they examined him themselves, they saw that he didn't need to be admitted. He could be given medication and go home, and return if needed. Although they tell you if any challenges arise at any time, you should bring him back. Don’t wait for the scheduled date, if a problem occurs, bring him to the hospital immediately. | family involvement, support from healthcare providers |
| INTERVIEW 2 ENGLISH VERSION.docx | At the family level, I am grateful. Together with the children, we have been together, along with relatives and friends. I am thankful for that. They have supported us materially and in other ways because during the time when he couldn’t do anything and needed to be lifted, I couldn’t do it alone. So, there were always people at home | family support |
| INTERVIEW 2 ENGLISH VERSION.docx | My suggestion is that, you know, these people with stroke face challenges. For example, when they arrive at the clinic, they should be served quickly and leave. You see, sometimes you find a person with a stroke sitting in the clinic for more than five or six hours. | stroke survivors should be prioritized |
| INTERVIEW 2 ENGLISH VERSION.docx | And as for the healthcare providers, because they are human beings too, sometimes you might encounter one who maybe woke up on the wrong side of the bed and might be a bit harsh, not using friendly language, especially for patients or elderly people like that. But one such person shouldn’t be seen as representative of the entire group that is doing a good job. | poor communication from healthcare providers |
| INTERVIEW 2 ENGLISH VERSION.docx | Maybe the government should look into how, because we hear, or I don't know if it's true, that in India, people get therapies to dissolve those clots. The government should prioritize that and bring in machines so that people can get treated, stand up, and continue with their activities. | government investing in stroke treatment |
| INTERVIEW 2 ENGLISH VERSION.docx | If there could be a way to get machines or drugs to dissolve these clots so that a person can be treated at nearby healthcare facilities and continue with their activities, that’s my advice | Ensure stroke medication availability at PHC |
| INTERVIEW 9 ENGLISH VERSION.docx | we were admitted at a district hospital. Later, we came to the city and went to the reginal hospital. She was treated there, and we were given a referral to come here. | referred to big hospital, transfer to big hospital |
| INTERVIEW 9 ENGLISH VERSION.docx | , that’s when she started receiving care here. I’m not sure of the exact date. then she started receiving care, the service was good, with no issues. They were very cooperative. We stayed for almost a month and a half, or until the second month was ending | care at big hospital, admitted for more than one month, good services, satisfaction with services, satisfied with care |
| INTERVIEW 9 ENGLISH VERSION.docx | She had started developing many sores | pressure sores after stroke |
| INTERVIEW 9 ENGLISH VERSION.docx | after being tested many times, they advised me to get tested too. I went for a test on the 18th, and they told me, “Sister, you are fine, continue to take care of yourself, and come back on the 18th of July. | caregiver health assessment |
| INTERVIEW 9 ENGLISH VERSION.docx | There is a need to spread education about stroke by using radio and television. Also the gen Z like social media, education can also be provided through those platforms so as to reach many people | Stroke education on radio and TV, stroke education on social media |
| INTERVIEW 9 ENGLISH VERSION.docx | One day, he said he felt weak, so we went to the hospital. That’s when his condition worsened; he couldn’t speak, his eyes were closed, and he couldn’t walk. | weakness after stroke, care at general hospital, loss of speech after stroke, vision loss after stroke, failure to walk after stroke |
| INTERVIEW 9 ENGLISH VERSION.docx | The challenges are, as you know, when you have a patient, your economy stops. You have to sell whatever you think might help with the illness. You might sell anything to help your patient. My brother-in-law suggested we bring him here, so since we came, my brother-in-law has been struggling, yes, he has been struggling, yes, his brother. I am his sister-in-law. | financial struggle after stroke |
| INTERVIEW 9 ENGLISH VERSION.docx | my brother-in-law and my uncle were the ones receiving the updates. There wasn’t a day they weren’t informed about his condition, how he was progressing, and what tests were done. They did many tests. | information sharing |
| INTERVIEW 9 ENGLISH VERSION.docx | After staying for so long and seeing his condition, we saw that they were doing their best, but we also saw that they had reached a point where they had done all they could. There were many drips, small drips, many injections, and many pills. They started to gradually reduce these and advised us to use the medication properly and come back for a check-up. We weren’t worried because they took good care of him. | satisfaction with services, satisfied with care |
| INTERVIEW 9 ENGLISH VERSION.docx | I would say that the care they provided to my husband was good, and they should continue with their good services. They did their best, but it’s up to God. They gave him exercises, but sometimes he couldn’t even stand or hold anything. They tried to stretch and help him | appreciations to healthcare providers, good services, satisfaction with services |
| INTERVIEW 10 ENGLISH VERSION.docx | When she had the stroke, I brought her, and we were received at the emergency, we were received well, and we were taken to the ward. She stayed in the ward for four days, they checked her and said it was high blood pressure, they controlled the pressure, but after that, she was not able to get up or anything | satisfaction with services, satisfied with care, good services |
| INTERVIEW 10 ENGLISH VERSION.docx | During the time she was in the ward, the time we came to see the patient, because you are not allowed to stay with the patient full time, so in the morning when I came to see my patient, after providing care like feeding and bathing her, I would go to the nurse station and ask the doctor about my patient’s progress. They would cooperate, take the file, and read to you about the patient’s condition. If the results were not ready, they would tell you to come at a certain time, and indeed, when you came at that time, you would get your results and meet the doctor who would explain your patient’s condition. | short visiting time, good communication with healthcare providers, support from healthcare providers, cooperation with healthcare providers |
| INTERVIEW 10 ENGLISH VERSION.docx | During the time she was in the ward, the time we came to see the patient, because you are not allowed to stay with the patient full time, so in the morning when I came to see my patient, after providing care like feeding and bathing her, I would go to the nurse station and ask the doctor about my patient’s progress | feeding by relatives, bathing stroke survivors |
| INTERVIEW 10 ENGLISH VERSION.docx | So, she was doing things that were not appropriate at that time, but the nurses took good care of her. I came and found they had taken good care of her. Even in the process of getting blood, I had not yet found enough people to donate blood, but they went and brought blood and said, “We cannot take care of her without first giving her blood while you are still looking for donors,” and indeed, I found donors and donated. | support from nurses, good services, satisfied with care, satisfaction with services |
| INTERVIEW 10 ENGLISH VERSION.docx | Regarding discharge, when I arrived, I was just called by the nurse at the nurse station, and she told me that your patient has been discharged. Wait for us to prepare the medication and the bill, and come to pick her up at a certain time | discharge notice given on the day of discharge |
| INTERVIEW 10 ENGLISH VERSION.docx | Honestly, I was not told that today and then tomorrow I would be discharged. I came in the morning and was told that at noon at 12, she would be discharged | unprepared discharge |
| INTERVIEW 10 ENGLISH VERSION.docx | In our family, we are three. The good thing is that we help each other. I am grateful that we help each other because I am the only girl, and my younger siblings are boys. In most cases, I participate more because my mother is a woman, and I am a woman. They help me with things like lifting her and such | collaboration among family members, gender issues in caring |
| INTERVIEW 10 ENGLISH VERSION.docx | Regarding transportation, it is really a challenge because the place where my mother lives to get here, if we take a bajaji (three-wheeler), it costs 25,000 TZS one way and 25,000 TZS to return, so it is 50,000 TZS. So, right now, things have been a bit tough because I spend most of my time nursing the patient and not working. | transport costs to hospital, stop working to nurse my mother |
| INTERVIEW 10 ENGLISH VERSION.docx | The biggest obstacle is transportation because for a patient like this, when you lack money to take a “bajaji,” you have to use public transport, especially the rapid buses. There are obstacles in transportation, but as a human being, I cannot say that due to obstacles, I failed to take my mother to get care. That has never happened. There are challenges, we encounter them, but we fight through them and ensure she gets care and sees the doctors as usual. | transport costs to hospital, transport to hospital |
| INTERVIEW 13 ENGLISH VERSION.docx | We had to spend a lot of time at the hospital, dealing with the patient. It’s hard to focus on other things when you’re constantly attending to their needs. | hospitalization is time consuming, hospitalization shift focus of other activities |
| INTERVIEW 13 ENGLISH VERSION.docx | Ah, the diagnostic challenges were significant. We struggled to get test results. Sometimes they’d say the CD was corrupted or missing, and we’d have to repeat the tests. On the first day, all we got was the CT scan, and it took three days to receive the full results. | Improve care communication, improve services |
| INTERVIEW 13 ENGLISH VERSION.docx | Well, it’s not just the doctors; everyone needs to keep an eye on the patient. Sometimes the doctor who ordered a specific test isn’t the one interpreting the results. So, you ask about the CD, and they tell you it hasn’t been read yet or that it’s missing. | Improve care communication |
| INTERVIEW 13 ENGLISH VERSION.docx | “No, we weren’t given any specific instructions. We just knew that her body was weak because she wasn’t eating properly. Eating has been a challenge for her.” | no discharge information, feeding is challenge |
| INTERVIEW 13 ENGLISH VERSION.docx | “No, we did ask. When the test was taking longer, we inquired about it. | asking questions to HCPs |
| INTERVIEW 13 ENGLISH VERSION.docx | “Well, there’s a phone number we wrote down. That’s where they send all updates about the patient. So, they kept us informed.” | updates given through phone calls |
| INTERVIEW 3 ENGLISH VERSION.docx | For a stroke patient, for example, you might tell them to get up at a certain time to take their medication because the medication needs to be taken in the morning and at night. So, by six in the morning, you need to be up, have prepared them, bathed them, and given them their medication | dedication in caregiving |
| INTERVIEW 3 ENGLISH VERSION.docx | The act of waking them up to give them medication is a task. Therefore, as a caregiver, you must be dedicated. If you are not dedicated and are lazy, it becomes a significant challenge. You might find yourself caring for a patient every day without seeing any improvement. | dedication in caregiving |
| INTERVIEW 3 ENGLISH VERSION.docx | Initially, it was difficult because we thank God that while he was admitted here, he was being cleaned and taken care of in every way. So, going home, we had to start afresh and take care of him ourselves. | worries of home care |
| INTERVIEW 3 ENGLISH VERSION.docx | I think on our part we received very good care at the tertiary hospital. We thank God for that because from how he came into how he left; we received very good care. | good services, satisfaction with services, satisfied with care |
| INTERVIEW 3 ENGLISH VERSION.docx | The services from the nurses, the cleaners, everything was fine | support from healthcare providers, support from nurses |
| INTERVIEW 3 ENGLISH VERSION.docx | We come to the clinic and for the physiotherapy. | attend clinic, attend physiotherapy |
| INTERVIEW 3 ENGLISH VERSION.docx | the challenge is just the queue, and sometimes you can come with a patient, and you can sit there with the patient, and sometimes people who are not sick, elderly people, are sitting in the chairs and they don't want to give up the seat for the patient, so you find yourself standing with the patient. | Long waiting time, improve services delivey |
| INTERVIEW 3 ENGLISH VERSION.docx | So, I mean, they should look at the seating area for patients and the seating area for elderly people because the elderly person is not the one who accompanied the patient, and then you come with a patient who can't even stand, and the person continues to sit in the chair, so that's the challenge | improve waiting area |
| INTERVIEW 3 ENGLISH VERSION.docx | The cooperation is very good, the cooperation is very good from the healthcare services to the exercises. The cooperation is very good. Firstly, they have done a great job with the exercises; they have a high level of cooperation and good advice. | cooperation with healthcare providers |
| INTERVIEW 3 ENGLISH VERSION.docx | The cooperation in exercises, when you get there, the exercises are done on time, you are advised not to do this, do this, do the exercises like this. When you get home, try to do the exercises as advised. They have good cooperation. | cooperation with healthcare providers, support from healthcare providers, exercise at home |
| INTERVIEW 3 ENGLISH VERSION.docx | there was a time when he refused to do exercises, so they called me and advised me to encourage him to do exercises, stating that exercises are crucial. Then I called him inside, we sat with the doctor, and he advised him, so in many aspects, they were involving me. | family involvement |
| INTERVIEW 3 ENGLISH VERSION.docx | They asked us where we lived, I said we live in an apartment, but given the current situation, the doctor asked if he would be able to manage the environment, and we said yes, he would be able to manage it. | good communication with healthcare providers, support from healthcare providers |
| INTERVIEW 3 ENGLISH VERSION.docx | Yes, I recommended. The doctor told us about physiotherapy, about carrying heavy things because, at home, he shouldn't just sit idly. So, we suggested that there are devices being sold, like electric ones, for the hand, so I suggested that such a device be bought. After I gave the advice, the device was purchased, and he uses it at home. | family involvement |
| INTERVIEW 3 ENGLISH VERSION.docx | About 80% of my recommendations have been listened to. | degree of involvement |
| INTERVIEW 3 ENGLISH VERSION.docx | No, I haven’t received any unclear information. All information was beneficial; there was no unclear information. | information was beneficial, good communication with healthcare providers |
| INTERVIEW 3 ENGLISH VERSION.docx | When the patient came from RRH to the reception here, we had to go for a test, so we had to go to here. Therefore, you had to hire an ambulance to go to one hospital and return. This is a challenge because not everyone can afford that. If a test is available at one hospital, why can't it be brought here? Because today, someone may have the means to hire an ambulance to take their patient to tertiary hospital, get the test done, and return, but another person may not have that ability. How do you help such a person? So, it’s a challenge for patients | availability of advanced stroke care, tests availability, distance for investigations, cost for services |
| INTERVIEW 3 ENGLISH VERSION.docx | My suggestion is that the regional and district hospitals need to be equipped with medications, stroke experts and investigations tools like CT and MRI, rather than having the patient go from district hospitals to tertiary hospitals, which are very far and expensive | Suggest to improve care at primary facilities |
| INTERVIEW 3 ENGLISH VERSION.docx | They should continue to provide more education, as caring for a patient is a learning experience. They need to keep educating us more. For example, when we bring patients, maybe the nurses who come daily should provide more education on how to live with the patient, what the patient does, and how to handle the patient. More education will help us in taking care of our patients. | training for family members |
| INTERVIEW 3 ENGLISH VERSION.docx | Yes, the doctor asked who would be staying with the patient when he was being discharged. I was called, and the doctor gave me the medication instructions. | caregiver involvement, medication instructions after discharge |
| INTERVIEW 3 ENGLISH VERSION.docx | Hmm... As for this hospital the services are good, I really like them. I am pleased, and I had never been to this hospital before; I came specifically for my brother. I had only heard about it, so the services are excellent than the services in our district and regional hospitals | satisfaction with services, satisfied with care, good services |
| INTERVIEW 11 ENGLISH VERSION .docx | When she had the stroke, we were admitted to a district hospital. | patient admitted in district hospital |
| INTERVIEW 11 ENGLISH VERSION .docx | Later, they said she needed a CT scan and should be taken to the regional hospital | referred to big hospital, patient did CT Scan |
| INTERVIEW 11 ENGLISH VERSION .docx | We were taken to the regional referral hospital | referred to big hospital |
| INTERVIEW 11 ENGLISH VERSION .docx | When we got there, they did a CT scan and found that one of the blood vessels in her head had burst, and blood had clotted | patient did CT Scan, patient has bleeding in the brain |
| INTERVIEW 11 ENGLISH VERSION .docx | They couldn’t treat her; they were just lowering her blood pressure | treated at regional hospital |
| INTERVIEW 11 ENGLISH VERSION .docx | After that, they referred us to tertiary hospital. They didn’t provide us with transport from the regional hospital, saying that since the patient was not critical, we had to use our own transport and expenses. I didn’t see this as healthy because if the patient is complaining of a headache all the time and the blood pressure is persistently high, and they tell you they won’t provide transport because the patient is not serious | referred to national hospital, didn’t use ambulance, unsatisfied with referral transport logistic, patient had headache after stroke |
| INTERVIEW 11 ENGLISH VERSION .docx | We were grateful that when we arrived, they received us very quickly and started treatment. They told us that the patient’s problem was as described in a regional hospital, but the doctors for this problem were at here at tertiary hospital. They provided us with transport to Tertiary hospital. Now I was wondering, does this mean she had become critical here. Because at tertiary hospital provided us with transport, but at regional hospital they said couldn’t because she wasn’t critical. I thank God that when we arrived here, they started treatment, and on the second day, the patient started showing improvement | good services, used ambulance at national hospital, better improvement after treatment |
| INTERVIEW 11 ENGLISH VERSION .docx | I prefer that if they refer a patient, they should handle everything, not just halfway, as it is not healthy | RRH should provide ambulance during referral |
| INTERVIEW 11 ENGLISH VERSION .docx | for stroke patients, travelling from far to the tertiary hospital with your own transport without a nurse or doctor is very dangerous because you never know what can happen on the way | used private transport, travelled without escort of HCPs |
| INTERVIEW 11 ENGLISH VERSION .docx | We didn’t have any equipment or medication, so you can’t know what the blood pressure is at any given time, and you can’t know if the car will break down. I thank God we travelled safely, but that was my challenge. | travelled without medical equipment or medication, patient condition can change during transit |
| INTERVIEW 11 ENGLISH VERSION .docx | Well… what I mean is that if a nurse is present, they are already trained in that problem. We might be in a situation where the blood pressure has risen, and they would know how to bring it down or if it has dropped too much, they would know what to give to bring it to the right level | need of a nurse during referral |
| INTERVIEW 11 ENGLISH VERSION .docx | we were received, but the challenge we faced was that we arrived at night, and until 5:00 AM, the doctors hadn’t started the medication yet. So, there was a bit of a delay in the doctor starting to attend to her. That was the challenge I found here, but after that, they attended to her well, and she started showing good results. | delayed treatment |
| INTERVIEW 11 ENGLISH VERSION .docx | the headache started to reduce because the main problem was severe headaches. The pain reduced, and she became more aware, even recognizing which hospital she was in. |  |
| INTERVIEW 11 ENGLISH VERSION .docx | I wasn’t sure if it was the procedure, but I would go directly to the doctor to get updates on the patient’s progress. The doctor would tell me that the patient had one, two, three issues but was improving because even her awareness was returning, and it was true | interactions with healthcare providers, good communication with healthcare providers, given feedback on pt progress |
| INTERVIEW 11 ENGLISH VERSION .docx | For example, on the third day, they told me that the patient needed to have her heart checked with two tests. I took her for the tests, and they told me that there were no issues found, so she had no other problems besides the stroke. | family involvement, sharing updates with family |
| INTERVIEW 11 ENGLISH VERSION .docx | Yes, it’s true. Even he was wearing a coat | identified a doctor by name and dress |
| INTERVIEW 11 ENGLISH VERSION .docx | They would often go to the patient when I wasn’t there and come back with updates that the patient was improving | given feedback on pt progress, information sharing |
| INTERVIEW 11 ENGLISH VERSION .docx | They would say there were no other issues, but I would tell them that although there were improvements, she wasn’t fully recovered. They said she would recover based on the current situation. I also told them that I still saw a problem with her eyes; she was seeing double. They said that issue would also resolve with continued medication, and we are waiting to see the results as they told us to return on the first. | interactions with healthcare providers, good communication with healthcare providers, benefits of inquiry, asking questions to HCPs, encouragements from HCPs |
| INTERVIEW 11 ENGLISH VERSION .docx | Ah, I think it is very important for the doctor in charge to be professional and to see the nurse directly. When information comes through the nurse, it changes. Some nurses don’t receive it as it is; they just take it superficially. So, I think it is very important that, if possible, even for a short time, the doctor should sit with the patient’s relative and give direct instructions. I think this is healthier than receiving second-hand information… like, “the doctor said this and that,” which is not very healthy. | doctors should give direct feedback to relatives |
| INTERVIEW 11 ENGLISH VERSION .docx | I think it would be better because when you get it directly from the doctor, the doctor says exactly what they saw. But with a nurse, they might leave out some things, or forget | doctors should give direct feedback to relatives |
| INTERVIEW 11 ENGLISH VERSION .docx | I was happy because if they involve you and tell you that the patient has one, two, three issues and needs to be treated in a certain way, it’s a good thing. | felt good to be involved in care |
| INTERVIEW 11 ENGLISH VERSION .docx | was involved in that they told me my patient’s blood vessel in the head had burst, causing the blood to clot, which was causing the problem. But I didn’t know what to do because they told me. I asked them what method we would use to remove the blood, whether it would be surgery or something else. But they told me that the condition would resolve on its own, which is what they answered. | family involvement, good communication with healthcare providers, interactions with healthcare providers |
| INTERVIEW 11 ENGLISH VERSION .docx | Yes, I don’t know what medicine they used. They told me the condition would resolve on its own with the blood pressure medication they were using. | inadequate information |
| INTERVIEW 11 ENGLISH VERSION .docx | I think healthcare providers should sometimes come to you directly. I don’t like having to go to them and say, “My patient has this and that.” It would be better if they came to you and said, “Your patient needs this medication or this injection for this reason.” You should know what they are treating. | HCPs should offer timely feedback, HCPs should offer direct feedback, HCPs should interact lively with caregivers |
| INTERVIEW 11 ENGLISH VERSION .docx | Right now, I am uncertain; I don’t know what medication they used to dissolve the blood clot in the head | improve feedback |
| INTERVIEW 11 ENGLISH VERSION .docx | Because we came on the second day, we were told she was given an injection here, blood was drawn here, and this was done, so we don’t know what medication was used. | improve feedback |
| INTERVIEW 11 ENGLISH VERSION .docx | The information I was given was reassuring. First, discovering the problem that the patient had blood clots in the blood vessels in her head was a relief. Knowing the illness gave me hope because if the problem is known, it is easier to treat. | information gives hope and assurance |
| INTERVIEW 11 ENGLISH VERSION .docx | I thank God that although I don’t know what medication they used, they treated her because I see the changes. | improve feedback |
| INTERVIEW 11 ENGLISH VERSION .docx | The changes I see are that she couldn’t walk by herself before, but now she can walk around the house even three times by herself. She couldn’t eat, but now she eats by herself. Before, her eyes were constantly moving, but now she looks normally, although she still sees double. I asked about it today, and they said that issue will resolve as she continues to recover. | improvement after treatment |
| INTERVIEW 11 ENGLISH VERSION .docx | On the first day of discharge, the nurse advised me to stay in the city for two weeks so that it would be easier to return on the fourth, rather than going directly to the region. I found that advice helpful because traveling a long distance at that time wasn’t suitable for her condition. So, we stayed here for two weeks, and today we returned | nurses advices helped to cut costs |
| INTERVIEW 11 ENGLISH VERSION .docx | There is a bit of a problem with stroke patients, as I have observed. They shouldn’t have too many visitors because talking too much causes headaches. There should be a limit, maybe two people a day, one at a time. But as you know, everyone wants to come and see her, and it’s hard to limit them. Sometimes, six, seven, eight, or even ten people come, and they all want to see her. But the patient shouldn’t have too many people around because noise and disturbances cause headaches. | stroke pts needs more time to rest, reduce number of relatives to visit pt |
| INTERVIEW 11 ENGLISH VERSION .docx | When I saw that the patient was having trouble, I tried to educate the relatives, and they understood. Later, they would call to ask about her condition, and I would respond over the phone. I also tried to organize it so that maybe one person would bring food each day, giving them a chance to see her. | caregiver support, family support, collaboration among family members |
| INTERVIEW 11 ENGLISH VERSION .docx | I think healthcare providers should sometimes come directly to the patient’s relative and give instructions. It’s better than having to go to them and ask about the patient’s condition. They should inform you directly about the medication or injections needed and why. Right now, I am uncertain about what medication was used to dissolve the blood clot in her head. There was no education provided. | doctors should give direct feedback to relatives |
| INTERVIEW 7 ENGLISH VERSION.docx | we took her to a regional hospital. she stayed there for a night, and the next day they said she needed a CT scan. They said to send money, which was sent, and she was scanned. After that, they told us that the patient had a burst blood vessel in her head, so they couldn’t handle her there, and she needed to be transferred to Tertiary hospital | care at general hospital, transfer to big hospital |
| INTERVIEW 7 ENGLISH VERSION.docx | There, they did more tests, and then she was transferred here, where she was examined again and admitted. | transfer to big hospital |
| INTERVIEW 7 ENGLISH VERSION.docx | She stayed here for about two weeks, and then they allowed her to return home, with a follow-up appointment set for today | discharged home, admitted for two weeks, attend clinic |
| INTERVIEW 7 ENGLISH VERSION.docx | Her arm was completely not paralyzed but now if we move it, she says it hurts, … leave me. So education on the importance of exercises is very important to ensure consistency and adherence to physiotherapy | physiotherapy is painful, tertiary education, rehabilitation education |
| INTERVIEW 7 ENGLISH VERSION.docx | For example, with the discharge, there was a bill, and they said it had to be paid before you could be discharged from the hospital. So, we had to wait that day because the money wasn’t available. The next day, people came together, and the money was found. We paid the debt, and then we were given the patient and left. | financial issues during discharge |
| INTERVIEW 7 ENGLISH VERSION.docx | Well, they said that because we came at different times, one person should take the patient’s reports and provide updates on the patient’s status. They didn’t want everyone to come and give their own updates. So, I registered my name, and then her husband would go and ask. He would get answers like if there was a test, you would get a paper to pay for it yourself. | sharing updates with family |
| INTERVIEW 7 ENGLISH VERSION.docx | On the day of discharge, they wrote a paper for us because my brother-in-law was handling it. I was downstairs. That day I was coming in the evening, and they said she was discharged, but there was a bill that needed to be paid before we could leave with the patient. That day, we didn’t have the money to pay the bill. The next day, my brother-in-law paid it, and then we left. | discharge notice given on the day of discharge |
| INTERVIEW 7 ENGLISH VERSION.docx | At that time, sometimes they would give her medicine, but she couldn’t take it. We would find her vomiting the medicine because she was stubborn about taking it. We asked if we could watch her, and they said they would take care of it. We asked to come and check on her, and they said the staff were there. So, we stayed downstairs and would stay there until night or my brother-in-law would sleep in the area with many people. | suggestions ignored, sleeping with patient in the hospital |
| INTERVIEW 7 ENGLISH VERSION.docx | Mmmnhh. Also, they said at a regional hospital that she had a burst blood vessel and bleeding in the brain, and she needed to be brought here. But here, I didn’t hear anything about surgery or anything. I didn’t hear anything about it, and they said it was dangerous if the vessel was burst. So, I didn’t continue to ask. My brother-in-law said she was just given medication, and she took it until the day we heard she was being discharged. We asked about surgery but didn’t understand further. | inadequate information, fear of inquiry |
| INTERVIEW 7 ENGLISH VERSION.docx | Mmmnhh. They were saying we needed to pay for tests. We would ask what tests were needed, and we would pay and get receipts. But I don’t know if he was told anything specific by the doctor because we were separated. | tests not done |
| INTERVIEW 7 ENGLISH VERSION.docx | Initially, we had to mash her food, like porridge, because she couldn’t chew properly. I said she should drink from a cup, and now she is able to chew. I don’t mash food like before; I just cook regular bananas, which she can chew. However, she still can’t eat meat, but she can chew fish. Initially, she couldn’t even handle bones, but now she can remove them herself. | changing food texture after stroke |
| INTERVIEW 7 ENGLISH VERSION.docx | Well, if they had educated us on what kind of care a patient with this condition should receive, like specific practices or exercises, it would have been helpful. Some patients might have difficulty speaking but can write down their needs or feelings. I haven’t received that kind of education yet, maybe we’ll get it today when we come. | discharge education, training for family members |
| INTERVIEW 7 ENGLISH VERSION.docx | Also, for patients who shouldn’t be isolated, I think it’s better if they are around people. Even if they say they’re tired and need to lie down, being in the company of others, hearing children play and other sounds, might help them. Initially, she wasn’t speaking, but now she says she’s tired and needs to be lifted. Sometimes, when her bed is near a window, she holds onto the bars to help herself sit up. | companion from family members, presence of family members |
| INTERVIEW 7 ENGLISH VERSION.docx | We placed a mattress on the floor to catch her if she falls because she still has balance issues on one side. We put the mattress there so that if she slips, she won’t fall and get hurt. | patient fall at home |
| INTERVIEW 7 ENGLISH VERSION.docx | neighbors came to visit the patient and would bring food. Others would say, "I'll take food today," and we had a system where only two people could be with the patient at a time. We would rotate, with some staying for a few minutes and then leaving so others could come in. Neighbors also visited, and staff members came to pray at home and even when the patient was in the hospital. | support from neighbors, support from church leaders |
| INTERVIEW 7 ENGLISH VERSION.docx | However, the challenge sometimes is not having transportation money to bring the patient here and take them back. You can’t put someone in a dala-dala (bus) with that condition because you need to support them physically. So, transportation can be a challenge. For example, a brother of ours said he would provide transportation and asked us to let him know the hospital return date. I told him the date was recent, but when I called him yesterday, he was traveling abroad. So, we were left wondering what to do. A neighbor offered their car and said they would drive us, so we brought the patient here. | transport to hospital, collaboration among family members |
| INTERVIEW 7 ENGLISH VERSION.docx | It’s education—knowing how to care for these people. | family members need education |
| INTERVIEW 7 ENGLISH VERSION.docx | Education on how to care for a patient like this—what needs to be done for them. | training for family members, family members need education |
| INTERVIEW 7 ENGLISH VERSION.docx | Just praying to God, asking Him to give us others because caring for a patient requires a lot of heart. Well, I try not to be too angry, even though the patient might be difficult, sometimes you call them and they might just be grumpy and not feeling well | care with love |
| INTERVIEW 7 ENGLISH VERSION.docx | You don’t know if it’s high blood pressure or something else until you bring them to the hospital. We don’t have a way to measure it at home; now they have devices that you can use at home. | blood pressure measuring at home |
| INTERVIEW 7 ENGLISH VERSION.docx | But sometimes it’s also a cost to bring them here and take them back. You might hear that they feel bad or their blood pressure has risen or fallen | patient change condition during clinic visit |
| INTERVIEW 7 ENGLISH VERSION.docx | What should we give them to stabilize their pressure? We need that education to know how to handle such situations. If you see they are very tired and weak, you need to know what their blood pressure is or if it has risen too high; we don’t know. We continue to give them medication because we were given a lot of medicine, and they said just give them food, grind it up if necessary. | inadequate information, discharge education, family members need education |
| INTERVIEW 7 ENGLISH VERSION.docx | When they’re a bit tired, we might mix in some juice or fruit or give them milk. We try to keep their mouth clean and stimulate them with conversation to help their memory a bit. If you had more education, you could guide us. | family members need education, discharge education, inadequate information |
| INTERVIEW 7 ENGLISH VERSION.docx | When he was discharged, it was written on a paper, it was in the afternoon. I found the paper and just thought it was for tests. I didn’t look into it; I was just taking care of my patient, feeding him, changing his diaper, washing dishes, and went downstairs. Downstairs I met his husband. I told him there was a paper upstairs. When he went up, we thought it was about medicine and that tests had to be paid for. His husband saw the paper and went to ask; he was told he was discharged but there was a bill that needed to be paid first. | discharge notice given on the day of discharge |
| INTERVIEW 7 ENGLISH VERSION.docx | we weren’t informed. We were only given the discharge notice on the same day; we found it there. His husband found it and said so. Since we came every time, in the morning, afternoon, and evening, I found it in the afternoon. In the morning, his husband didn’t come; someone else came, my other sister. I don’t know if he didn’t check. So, when I told him that day, he came and went upstairs to get the paper and went to ask. He was told that the patient was discharged, but there was a bill that needed to be settled. Maybe he knows what they told him. | discharge notice given on the day of discharge |
| INTERVIEW 7 ENGLISH VERSION.docx | For me, I met the doctor not long ago, but he told me about the progress and said it wasn’t going badly. However, he mentioned that another doctor would give further instructions. There was a smaller, younger doctor who said he would come but continued attending to another patient. | good communication with healthcare providers |
| INTERVIEW 5 ENGLISH VERSION.docx | That's when she called me, "Aunt, come." When I went there, I found her and asked, "Mama, what's wrong?" She told me, "I don't know, my hand feels heavy, and my leg feels heavy." I looked at her face and saw that her mouth had gone to one side, so I tried to straighten her out, and I managed to fix her mouth. Thankfully, her mouth returned to normal, but her hand and leg remained the same | unaware of stroke signs, weakness after stroke, mouth affected after stroke, left side stroke |
| INTERVIEW 5 ENGLISH VERSION.docx | I called my relatives, and they came. We just prayed, asked God for help, and called a Shekhe to pray for her. | family support during stroke onset, religious support during stroke onset |
| INTERVIEW 5 ENGLISH VERSION.docx | Then we took her to the regional hospital. She was given an injection—one injection—and placed on medication under her tongue. We stayed until evening, then we returned home. | survivor sent to hospital |
| INTERVIEW 5 ENGLISH VERSION.docx | one to massage her. After some time, she started walking, and she said, "This is enough, the rest is just exercise." But even after seeing that, we still went to a regional hospital for her clinic, for her blood pressure check-ups and exercises. | exercise at home, exercise is key, exercises at nearby hospital, blood pressure measuring at home |
| INTERVIEW 5 ENGLISH VERSION.docx | But the challenge is the cost of transportation from home to the hospital, which is a burden for me. | transport costs to hospital, transport is expensive |
| INTERVIEW 5 ENGLISH VERSION.docx | I can manage, but for the exercises, she needs to come here three times a week | physiotherapy 3 times weekly |
| INTERVIEW 5 ENGLISH VERSION.docx | Hiring a car from our home to here, if you request a taxi, it costs 24,000 shillings. I requested 24,000 shillings; going and returning costs 48,000 shillings. And my insurance doesn't cover this, so I have to pay cash | transport is expensive, insurances does not cover transport |
| INTERVIEW 5 ENGLISH VERSION.docx | I'm thankful that here at the doctor's, my insurance is accepted, but it doesn't cover the exercises | insurance does not cover exercises |
| INTERVIEW 5 ENGLISH VERSION.docx | That's the challenge, and there are many small issues that sometimes make me feel overwhelmed. She's my mother, and sometimes she can't walk, or sometimes she can stand up and fall. | feeling overwhelmed, patient fall at home, patient can not walk |
| INTERVIEW 5 ENGLISH VERSION.docx | Sometimes I'm busy, and I'm the only one there, so it's like that | caring alone at home |
| INTERVIEW 5 ENGLISH VERSION.docx | You just have to help stretch them out, do this and that for them. Sometimes you tell them to do it themselves, or sometimes you help them with the exercises | helping survivors with exercises |
| INTERVIEW 5 ENGLISH VERSION.docx | The main issue we face is money. Sometimes the insurance doesn't cover some places, or it doesn't cover certain medications. If they could consider elderly people or those who are less fortunate, it would help because we want to get treated, and you want your parent to get better | financial struggle after stroke, insurance doesn’t cover some medications, give priority to elders treatment |
| INTERVIEW 5 ENGLISH VERSION.docx | But as I mentioned, I can't bring her to exercises every day because I have to pay for them. When you go to the exercises, you have to pay. They say it's 5,000 shillings to see the doctor, and then there’s another fee you have to pay. In short, when I leave home to the hospital, I need to have 50,000 shillings on hand, which includes 50,000 for transportation. There's a bajaji that we have requested, which charges us 30,000 to go and return, and the remaining 20,000 is for the exercises. | transport costs to hospital, transport is expensive, cant afford physiotherapy |
| INTERVIEW 5 ENGLISH VERSION.docx | The doctor has advised that she needs to do the exercises regularly, three times a week, but from our home to here at Tertiary hospital is a challenge. | physiotherapy 3 times weekly, worried about 3 times physiotherapy costs |
| INTERVIEW 5 ENGLISH VERSION.docx | . Maybe I should go to regional hospital. I did go to regional hospital for the exercises, but I noticed that regional hospital doesn't have the same equipment as here. At regional hospital, they mostly just use electricity, and they only apply electricity therapy | Unsatisfied with physiotherapy at RRH, improve physiotherapy at regional hospital |
| INTERVIEW 5 ENGLISH VERSION.docx | That's why I prefer it here because they have bicycles, leg exercises, hand exercises, and they also stretch her, and sometimes they use electricity therapy too. But the distance to get here is what makes it difficult for me. | prefer physiotherapy at national hospital than RRH, improve physiotherapy at regional hospital |
| INTERVIEW 5 ENGLISH VERSION.docx | The doctor advised us on her diet to prevent her sugar levels from rising, so she doesn’t end up having both a stroke and diabetes. There are foods she's been told not to eat and others she should eat to control the sugar level. The doctor is really doing his best. I'm grateful to God, Alhamdulillah, they are trying their best. | support from healthcare providers, good communication with healthcare providers, family involvement, diet instructions during hospitalization, diet instructions after discharge, appreciations to healthcare providers |
| INTERVIEW 5 ENGLISH VERSION.docx | Ah, I haven’t seen people like you here, honestly. I’ve never had anyone ask me questions like this. When I come, I just go to the doctor, get the service, and then leave. I haven’t seen a suggestion box or anyone asking me questions like this. | unaware of suggestion box location, never to make suggestions of care |
| INTERVIEW 5 ENGLISH VERSION.docx | : I did the test like... yes, it was on a Monday, then we rested on Tuesday, and on Wednesday, we went to pick up the results and then went straight to the doctor; so, that’s when he told us, saying that her stroke had hit twice. The first time, and now the second time, he saw a scar on her head. | second stroke, stroke revealed after test |
| INTERVIEW 5 ENGLISH VERSION.docx | It had hit twice, so he advised her not to stop taking the medication because if she stops, the third one could hit, and it would be even worse. That’s why we come to the clinic every time her medications run out. | adhere to clinic, adhere to medications, worry of third stroke |
| INTERVIEW 5 ENGLISH VERSION.docx | honestly (Aaah), you get startled because, you know, a hospital is a hospital, a hospital is a hospital, and being transferred to a place you don’t know.. | worries of transfer to national hospital |
| INTERVIEW 5 ENGLISH VERSION.docx | I knew Tertiary hospital where you end up being moved around. So, even here, I had never been here before; I only came for my mother, so I was really startled, thinking I don’t know how I’ll be received, I don’t know if I’ll be moved around like they do at Tertiary hospital. | worries of care at national hospital, improve care at tertiary hospital |
| INTERVIEW 5 ENGLISH VERSION.docx | But I was received well. I was just moved around a little downstairs because of the referral letter, but once I got here, I had no issues, honestly. I was received well and shown to my doctor, and until today, whenever I come and mention a particular doctor, I’m taken directly to that doctor. | care at big hospital |
| INTERVIEW 5 ENGLISH VERSION.docx | I haven’t been given any confusing information, except for being told that my insurance doesn’t cover certain medications. That was really shocking because you rely on insurance to get your medications, and now you’re told it doesn’t cover some medications. You end up having to pay out of pocket because the insurance has declined. So, you find that sometimes you’re prescribed medication, and you have to go find it yourself. Given our situation here in Tanzania, sometimes when you come to the hospital and are told there’s a medication you need to go find, you have to struggle to find it. So the insurances should be comprehensive enough to cover all aspects of stroke care | insurance doesn’t cover some medications, no confusing information, insurance coverage is confusing |
| INTERVIEW 5 ENGLISH VERSION.docx | She has many children, but right now, it’s just me taking care of her, just making do with whatever little we have. I’m grateful that our father left us a house, so when we get some rent money, we use that to struggle with hospital expenses. | get money from rent house |
| INTERVIEW 5 ENGLISH VERSION.docx | But now, it’s like what they say: when an illness lasts long, people start focusing on their own families. The ones who were close to you are the ones you see enduring all the struggles, you see it all, and it’s you who ends up having to manage everything. When she faints, or something happens, I’m the one who feels worried, thinking about how my mother will be, where this will lead. | left alone because of illness lasting long |
| INTERVIEW 5 ENGLISH VERSION.docx | There are people who do the massages. They come with their oils and massage her. They massage the nerves, and it feels like there are small lumps or something like small bumps that they press out. They massage her leg and arm, and there are these little knots that used to be there at first, but after they massaged her, they went away. That’s what we call “kuchua” in the local Swahili slang. | massaging survivors |
| INTERVIEW 5 ENGLISH VERSION.docx | I think the main obstacle here is just money. If we had the money and could bring her for exercise regularly, I think she would be doing well. It’s really just about the exercises | financial issues affect clinic visits |
| INTERVIEW 5 ENGLISH VERSION.docx | If we had the means to get her here regularly or the transportation to bring her here often, that’s the main challenge. Transportation is the biggest problem. If I had transportation, I would bring her here regularly, even every day. | transport affect clinic visit, transport affect physiotherapy |
| INTERVIEW 5 ENGLISH VERSION.docx | I believe the exercises would help with the weight gain and numbness. So, if she could do exercises, I think she would be fine. Even though she’s taking medication, without the exercises, it’s not enough. If she could get regular exercises, she would be doing well. The biggest problem is transportation. | physiotherapy is key for recovery |
| INTERVIEW 5 ENGLISH VERSION.docx | I’m not sure about the improvements, but I feel like there should be a dedicated exercise facility, but it shouldn’t be far away. That’s the only thing I feel because I can say it’s far from here, but others may be close by. So, I’m saying it shouldn’t be far away. Maybe they could build a specific exercise center, but it shouldn’t be too far. Here, there’s good exercise equipment, but it’s the distance that’s the issue. If there were a specific place, we would know that this is just an exercise hospital. If you come there, you know it’s an exercise area, but it shouldn’t be far away like this. Even Tertiary hospital might be better, but I’m not sure if they have it there; I want to go and try Tertiary hospital, see if they can help. But if there were a dedicated exercise center, it would make it easier for people coming from far to Tertiary hospital. We would go directly there without having to enter the hospital; we’d just go straight to register for the exercises. The people there would only be focused on exercises. But maybe these are just my thoughts (Hahaha). | suggest to build more physiotherapy centres |
| INTERVIEW 5 ENGLISH VERSION.docx | I’ve never attended or maybe, maybe during the exercises, maybe you enter and sit for something, because I know that in the maternity clinic, you might sit in a class for about five minutes, or in the children's clinic, you might sit in a class. But I haven't received that here, or during the exercises. Right now, I just came from the exercise area; we did the exercises, then came up here to the clinic. So I haven’t received it, and I won’t lie and say that I have. I honestly haven’t received it. | not received caregiver education |
| INTERVIEW 5 ENGLISH VERSION.docx | The cause of stroke was high blood pressure, but we didn’t know about this illness. So I think there is a need to pass this education to the community through community programs and encourage people to check their health regularly. | unaware of stroke risk, encourage community drives, encourage screening for stroke |
| INTERVIEW 5 ENGLISH VERSION.docx | At Tertiary hospital clinic, I heard that in the Tertiary hospital clinic, we would sit in a class, and then they would teach us, they would teach about diabetes and high blood pressure. So that’s what I heard, that when blood pressure gets too high, it causes a stroke, or when blood sugar gets too high, it causes a stroke. | stroke education given in some centres |
| INTERVIEW 5 ENGLISH VERSION.docx | My biggest problem is with the exercises; when you think about the transportation cost and the cost of paying for the exercises, you find it difficult. So, they should consider the issue of exercises. So | financial issues affect clinic visits |
| INTERVIEW 5 ENGLISH VERSION.docx | if you show you need assistance, they usually have that exemption for the elderly, they treat you. But here, I haven’t seen anything like that, I don’t know if there’s any exemption. Some people advised me to go down there and ask, but I haven’t seen any place offering exemptions | exemption waiver for elders |
| INTERVIEW 6 ENGLISH VERSION.docx | I was put on leave at work because of the challenges my parent faces, as their condition has been fluctuating—one day they are like this, the next day like that. It reached a point where I had already taken my second leave, and then my employer forced caregiving leave to take care of my mother | took a leave, forced leave |
| INTERVIEW 6 ENGLISH VERSION.docx | We've been hospitalized no less than four times here, yes, admitted and discharged repeatedly. | readmitted several time |
| INTERVIEW 6 ENGLISH VERSION.docx | For instance, she now appears to have a deficiency in electrolytes, not to mention the stroke, and she seemed to be getting very tired. | tired after stroke |
| INTERVIEW 6 ENGLISH VERSION.docx | So, what we discovered was that when I asked the doctor who was treating her—because the first time we were admitted, we were given a lot of medication that the patient was using | discharge medications |
| INTERVIEW 6 ENGLISH VERSION.docx | She was told to take these blood pressure pills every day, one tablet. | adhere to medications |
| INTERVIEW 6 ENGLISH VERSION.docx | So, we were doing that, following the instructions, but then she started getting very tired | adhere to medications, tired after stroke |
| INTERVIEW 6 ENGLISH VERSION.docx | The result was that we had to bring her back for hospitalization | readmitted several time |
| INTERVIEW 6 ENGLISH VERSION.docx | We were given the same medication again, but finally, on the third admission, the last doctor said that she had low blood pressure. We informed the doctor that she had previously been diagnosed with high blood pressure and was prescribed medication for it. We even showed the medication we were given, and he suggested stopping the medication temporarily to monitor her condition. The result was that after stopping the medication, her blood pressure began to stabilize within a week without taking those pills. | medication reconciliation |
| INTERVIEW 6 ENGLISH VERSION.docx | On the last day we brought her here, it was a clinic day, and we gave her the blood pressure medication in the morning because we knew it was her clinic day. | adhere to medications, attend clinic, support from healthcare providers, poor communication from healthcare providers, inadequate information |
| INTERVIEW 6 ENGLISH VERSION.docx | That's what I've learned from caring for my relative: that now I know how to manage blood pressure. That's why I always ask what the medication is for and when we should administer it. | learning from caregiving |
| INTERVIEW 6 ENGLISH VERSION.docx | Anyway, they measured her blood pressure, gave her some medication | medication to dissolve clots, discharge medications |
| INTERVIEW 6 ENGLISH VERSION.docx | Anyway, they measured her blood pressure, gave her some medication, and we went back home. | discharged home |
| INTERVIEW 6 ENGLISH VERSION.docx | That same day, the stroke occurred because when we got home, we sat her down on a chair, and we were talking to her as a family. I called my older brother and told him, "Come here because our mother's condition isn't good." He came, and as we were talking, she mentioned that she wasn't feeling well. | feeling unwell after stroke |
| INTERVIEW 6 ENGLISH VERSION.docx | We need community-based education about stroke because majority of us don’t know. Also, primary care facilities can provide screening for blood pressure, sugar and fat so that we know our health. We caught her and laid her down on the bed. We saw that she was not herself; we thought maybe it was her blood pressure, so we gave her the medication she had been prescribed. After she took the medication, she slept. When she woke up, her arm and leg were paralyzed. | family support, unaware of stroke risk, unaware of stroke signs, paralysis of both hands after stroke, paralysis of legs, screening for health, community programs for stroke |
| INTERVIEW 6 ENGLISH VERSION.docx | When we got here, after she had the stroke, we went to a general hospital, and with the usual procedures, we explained the situation, and they | care at general hospital |
| INTERVIEW 6 ENGLISH VERSION.docx | She had to start physical therapy due to her condition. We were scheduled for therapy sessions here. | attend physiotherapy |
| INTERVIEW 6 ENGLISH VERSION.docx | The doctors saw her and scheduled therapy sessions to help her regain her normal functions | attend physiotherapy |
| INTERVIEW 6 ENGLISH VERSION.docx | Oh, sorry, before that, when her blood pressure was high, we were admitted to a private Hospital, where they monitored her heart. Her condition wasn't very good at that time, but she received treatment, and we were sent home. | care at general hospital |
| INTERVIEW 6 ENGLISH VERSION.docx | We reported to the OPD, and they told us to start the therapy sessions here. They gave us the schedule for the therapy sessions, and we brought her every Wednesday, Thursday, and Friday for the exercises. | attend physiotherapy |
| INTERVIEW 6 ENGLISH VERSION.docx | She was improving with the therapy, and we continued giving her the medication prescribed by the doctors | better improvement after treatment, adhere to medications, family support |
| INTERVIEW 6 ENGLISH VERSION.docx | However, even though we were giving her the medication, she seemed to be getting weaker | no improvement after medication |
| INTERVIEW 6 ENGLISH VERSION.docx | When she showed signs of needing to come back to the hospital, we brought her here. When you bring her back, they admit her and give her a bed | knowing the danger signs |
| INTERVIEW 6 ENGLISH VERSION.docx | Eventually, her condition reached a point where she could no longer do the exercises. We were told to continue doing the exercises at home just to keep her limbs flexible because she couldn’t walk or use a stationary bike anymore. | exercise at home |
| INTERVIEW 6 ENGLISH VERSION.docx | One of the doctors told us that we had to accept her condition and age. The best we could do were stretching exercises and other gentle movements. So that's what we did. Since then, even the therapy has stopped, and now we are in this situation. | good communication with healthcare providers, information about exercise, told to accept the situation because of age |
| INTERVIEW 6 ENGLISH VERSION.docx | Honestly, I can't lie—there was nothing but good service here at tertiary hospital. They received us well, and I didn't encounter any challenges that upset me. If you follow their instructions, things go smoothly. As for the services, although I don't have much experience, every time I came here, there were no problems | good services, satisfaction with services, services received in the ward, support from healthcare providers, good communication with healthcare providers |
| INTERVIEW 6 ENGLISH VERSION.docx | There was a time when the patient was in a bad condition, but we were discharged to go home. We were left wondering what to do since she was in that state. The doctor said there was nothing else but to care for her at home, focusing on medication and feeding her as instructed. So we had to manage her care in that condition. We had to accept it because the doctor had said so, and we returned home with that understanding. | home care is scary, worries of home care, premature discharge, patient condition during discharge, discharged home, discharge instructions given, medication instructions after discharge |
| INTERVIEW 6 ENGLISH VERSION.docx | When people came to see her, they would remark on how bad her condition seemed, but for those of us who stayed with her, we felt like she was doing better, even though in reality, she wasn't improving. She remained in the same state—no significant recovery, just maintaining her condition. She couldn't move on her own; we had to turn her and reposition her constantly. So, we did our best with the instructions we were given, but it was clear that her condition wasn't getting better; it just stayed the same. | support from neighbors, accepting the situation, no improvement after medication, caregiving burden, adhere to instructions |
| INTERVIEW 6 ENGLISH VERSION.docx | Ah, the cooperation in nursing is mainly while you're still here at the hospital. Once you leave the hospital, you just have to do what they've instructed you to do, which is difficult for short time to master. But they can visit us at home and teach us more. | services received in the ward, cooperation with healthcare providers, worries of home care, did not contact healthcare providers, need for home visits |
| INTERVIEW 6 ENGLISH VERSION.docx | But here at the hospital, they give you the medications and explain how to use them for the patient and how to manage their diet. After that, we take a “bajaji” (motorcycle taxi), put her in, and take her home. But beyond that, everything is completed here at the hospital | did not contact healthcare providers |
| INTERVIEW 6 ENGLISH VERSION.docx | Ah, being involved is important because if they hadn't involved me, then I would have ended up doing something wrong by now. | feelings of involvement in care |
| INTERVIEW 6 ENGLISH VERSION.docx | That hasn't happened. I've never been told to make decisions about something or anything like that; no, I've never been involved. | not involved in care decisions |
| INTERVIEW 6 ENGLISH VERSION.docx | There was a time when my mother was admitted here at the hospital, but I wasn't getting updates on her progress until I went to the reception and asked, "What's going on with my patient? I see she has a drip every day, but what's really bothering her, and what’s her status?" They would tell me to wait for the doctor to come and give me an answer. | poor communication from healthcare providers, inadequate information, dissatisfied with nursing care |
| INTERVIEW 6 ENGLISH VERSION.docx | One day, it seemed like they were misleading me; they said they were waiting for some tests or something like that. So I asked about those tests and mentioned that she was swelling. One of her arms was swelling, and when you pressed it, it felt like a mango. I asked them why she was swelling, and up to that point, I didn't understand. They told me there were some tests that had just been taken, but the results weren't out yet. The next day, it was the same thing. I was there, and I asked again, "Why am I not getting a clear answer?" Eventually, I insisted on speaking with the doctor directly to get a proper answer. Fortunately, I met the doctor, and he explained that they were checking her heart rate and other things. The results came back showing that her heart rate was fine and everything was okay, meaning the swelling was just a symptom on the side where she had the issue. | benefits of inquiry, good communication with healthcare providers, poor communication from healthcare providers, told that they were checking her heart rate and other things, inadequate information, |
| INTERVIEW 6 ENGLISH VERSION.docx | Well, from my experience, being discharged and going home feels like just a routine process. Once you get there, they tell you your patient has been discharged. You receive the discharge papers, and if there are any costs, you are required to pay them. They give you a control number, you pay, then you take the discharge papers to the reception, and they release you to go home. | discharge notice as routine |
| INTERVIEW 6 ENGLISH VERSION.docx | The education I received was like this: they told me that my patient should eat liquid foods because they are being fed through a tube, like thin porridge, which is what they said to give during certain periods because liquid foods are absorbed quickly in the body. So, it means they need to be fed in the morning, at 10:00 AM, in the afternoon, at 4:00 PM, and at 8:00 PM. | diet instructions after discharge |
| INTERVIEW 6 ENGLISH VERSION.docx | Education on medication... now, about education on medication, that’s where I faced a challenge. We weren't given any education on medication. You are given the medication but not educated about it. As I mentioned before, there was a situation where we were giving blood pressure medication while the patient's blood pressure was fine, and it ended up dropping. There’s no education here, right? I explained that there’s no education here, meaning I could have lost this patient because I didn’t know that you shouldn’t give blood pressure medication all the time, especially when it’s not needed. We were initially given over seven medications, but now it’s down to two, one for seizures and one for adding salt to the body. But before, the last time, I was given more than 14 medications; I would leave with a bag full of them, and she would take seven in the morning and seven in the evening. But it wasn’t clear whether these medications were for conditions that they had diagnosed themselves. You know, I’m not a doctor | limited discharge medication information |
| INTERVIEW 6 ENGLISH VERSION.docx | Or today, I see they prescribed the same two medications again, along with that tube for blood transfusion. Maybe there’s another challenge that might... | inadequate information |
| INTERVIEW 6 ENGLISH VERSION.docx | I mean, you come here, and they tell you, "He’s been discharged, go ahead and leave." There’s none of that here. The day I was feeding the patient, I didn’t even know they had been discharged. Someone came in the evening and told me that they had been discharged since noon. | discharge notice not given in advance |
| INTERVIEW 6 ENGLISH VERSION.docx | So, that day they had to spend the night because I was preparing to find money for transportation. That has happened twice now. Even the other day, they were discharged at noon, and I only found out when I came in the afternoon to feed them, but I wasn't informed. Someone who came to see the patient in the evening told me that the patient had already been discharged, and I was only informed at 4:00 PM. I wasn’t prepared, so we had to struggle to find money for transportation. We only managed to take the patient home late in the evening due to transportation issues | unprepared discharge |
| INTERVIEW 6 ENGLISH VERSION.docx | Honestly, there’s none of that here. They should call the person whose phone number they have on file to inform them that their patient has been discharged so they can prepare to come and pick up their patient. | unprepared discharge, a prepared discharge |
| INTERVIEW 6 ENGLISH VERSION.docx | I’m told there’s no problem, so we should leave. What else can I say? I mean, personally, until I’m told to leave, that they’re eating through a tube, meaning the patient should leave the hospital when they’re in good condition. Now, when they say to leave, the patient still has a catheter, so what can I say about that? (laughing) I just have to accept it because I’m not a doctor, and I don’t have that expertise. I’m told that the patient is okay and that we should go home and continue the care routine there | fear of inquiry, inadequate information, accepting the situation, patient condition during discharge |
| INTERVIEW 6 ENGLISH VERSION.docx | ’m very grateful to my family because we’ve been together in caring for our mother. I live with other family members, one of whom is my brother, and I’m the one staying with him, while the other siblings are elsewhere. | family support |
| INTERVIEW 6 ENGLISH VERSION.docx | you know, even though our mother is a Christian and we are Muslims, when people from her place of worship come to pray for her, they do so | support from church leaders |
| INTERVIEW 6 ENGLISH VERSION.docx | My wife is also there, helping in one way or another because she’s a woman, and there are some areas where I can’t reach, so it’s necessary for women to be involved. But for the parts within my capacity, I call my relatives, and they come, and we collaborate. | gender issues in caring |
| INTERVIEW 6 ENGLISH VERSION.docx | We start to handle things together, you see. We’re all men because we left the others at home, and we’ve told them to leave the rest to us. When it reaches a point beyond our capability, then they will have to step in. That’s how it is, but I’m grateful for that | family support, collaboration among family members |
| INTERVIEW 6 ENGLISH VERSION.docx | Although I’m currently facing challenges with employment, I don’t know if I’ll have a job or not, but I can’t leave my parent in such a situation. | worries about employment, caring is loving |
| INTERVIEW 6 ENGLISH VERSION.docx | Not really, she’s receiving care because she has insurance and everything, so she’s getting all the services she needs. The only challenge is if something isn’t covered by the insurance, we as a family have to find a way to help | health insurance, struggle on services not covered by insurance |
| INTERVIEW 6 ENGLISH VERSION.docx | I think their clinics should have a bit of a preferential treatment because this condition is becoming more and more prevalent now | stroke affect many people, preferential treatment for stroke survivors |
| INTERVIEW 6 ENGLISH VERSION.docx | I think their clinics should have a bit of a preferential treatment because this condition is becoming more and more prevalent now. When you realize that someone has had a stroke, I’m not sure what exactly is happening now | stroke is complicated disease, improve follow up care |
| INTERVIEW 6 ENGLISH VERSION.docx | So, I think the government should start looking at the actual state of these services, improving them, and figuring out what to do. | government invest in stroke care, government to figure out stroke burden |
| INTERVIEW 6 ENGLISH VERSION.docx | As for the nurses, it’s like this: they should have more compassion. Not all of them do; that’s why I said some people, as you asked me earlier, how do you get reports when your patient has been discharged, but you don’t even know they’ve been discharged? You find your patient with a challenge, like a dirty bed sheet, and someone just looks at it until you have to tell them, "This bed sheet needs to be changed." They say, "We'll come and change it," even though they’re making rounds to distribute medicine. So you end up having to do that job yourself. You go to ask for a bed sheet, and they tell you they’re out until later when the round passes by. So there are things that need attention | dissatisfied with nursing care, nurses need compassion, unclean patient surroundings |
| INTERVIEW 6 ENGLISH VERSION.docx | I think nurses should be educated about how to... for those who are being discharged and going home. Not all of them provide that education. Sometimes, we learn things from fellow patients who are also admitted there until you get the... there was a time when someone was admitted with their mother, and they told me, "You know, if they lie in these beds for too long, they develop sores." You understand? They told me there are certain mattresses that are sold, so I went and got one like you see here. They told me about a cream, so I went and bought it to apply on them, and now the sores have dried up. So, this is the kind of education we need to be given. You find that sometimes the patient lies down in the morning and stays like that until 4 p.m. or even until the next morning without moving. What do you expect? But at least at home, you can... now if they’ve been lying down for too long, you can turn them over like this... | nurses need to be trained on discharge care, inadequate information, peer interactions, peer education, dissatisfied with nursing care |
| INTERVIEW 15 ENGLISH VERSION.docx | “Certainly. One challenge was related to the initial care in the Emergency Department. Although we arrived within two hours, they didn’t provide the recommended antihypertensive medication promptly. There was a gap in understanding the urgency. Later, during the neurological consultation, they explained that it was too late for that specific treatment. | delay in receiving proper treatment |
| INTERVIEW 15 ENGLISH VERSION.docx | “Certainly. In the Emergency Department (EMD), we were informed about the need for early consultation. However, even within the department, there were delays. We had to wait for consultations with senior doctors. | delay in consultation with senior doctors at EMD |
| INTERVIEW 15 ENGLISH VERSION.docx | In the ward, there was a situation where the patient’s condition worsened suddenly. Within two hours, she couldn’t move her mouth or lift her arm. I was concerned. When I alerted the neurologist, they adjusted the treatment. Initially, we weren’t given the right medication, but later, they corrected it. | unsatisfied with ward care, unsatisfied with ward treatment, felt given inappropriate medication, patient condition worsened in the ward |
| INTERVIEW 15 ENGLISH VERSION.docx | For example, when I arrive and find that they have already explained everything to the patient, and since the patient is not medical personnel, when I ask them, they say, "I don't know what they said," they don't understand. | HCP gave feedback to patient, patient cant understand medical information |
| INTERVIEW 15 ENGLISH VERSION.docx | when we arrived at the EMD, they told me it was a TIA, not a stroke. They said, “So, we’ll discharge him soon.” It was 2 AM, and it was just me and my father. I thought, if I take him home, he can’t walk, and he’ll need to go to the washroom—how will I manage? So, I had to tell them, "I don't feel comfortable taking care of him at home just yet; let's stay for observation, and then you can discharge him if his condition improves." So, we were given observation. | suggested the patient to be admitted |
| INTERVIEW 15 ENGLISH VERSION.docx | Mhm, they came, did the rounds, talked to us, and explained the care plan and everything we needed to do at home, like physiotherapy. The physiotherapist came and explained the exercises, gave us follow-up dates, provided the medication, and then we were discharged. So, we left in good spirits. | discharge education, discharge instructions given, discharge medications |
| INTERVIEW 15 ENGLISH VERSION.docx | We were educated on diet; they explained the diet to us, the physical activity, the follow-up, and the medication. On that day, they also explained how his illness had progressed. | diet instructions after discharge, discharge medications, attend physiotherapy |
| INTERVIEW 15 ENGLISH VERSION.docx | The feedback was good because for the tests, even when I wasn’t there—my mother or siblings were there—when I came back, I would find some answers. For instance, they would tell me, “We were told a certain artery is blocked,” so there was feedback on the tests, | given feedback on pt progress |
| INTERVIEW 15 ENGLISH VERSION.docx | We didn’t really understand the feedback because we were told by the doctor to lie down there, so we did the test, but they didn’t tell us the results. Later, they said, “I’ll send them to you on the computer”. I’m not sure if the doctor was tired or what, but I didn’t really get an explanation; otherwise, everything else was okay. | inadequate feedback, inadequate information |
| INTERVIEW 15 ENGLISH VERSION.docx | There was something—I'm not sure if it was carelessness on the part of the Radiology team. We were told they were taking him for an MRI and MRI angiography. So, he went, came back, and the patient said they had inserted contrast into his arm. The next day, in the afternoon, they told us he would be discharged in the morning. Now, it's afternoon, and I’m following up on the discharge, and I’m told the patient hasn’t been discharged because he hasn’t done the MRI angiography. But he did the angiography yesterday! | miscommunication at radiology department |
| INTERVIEW 15 ENGLISH VERSION.docx | So far, this is the first follow-up; yeah, and during this one, we saw the doctor, and it seemed like we were maintaining the dosage. But when we went to the pharmacy, we were given a quarter of the dose, so I don’t understand—did the pharmacy make a mistake, or did the doctor overlook it, or did they really change the prescription? | inadequate feedback, inadequate information |
| INTERVIEW 15 ENGLISH VERSION.docx | The support has been great—mhm , I have relatives at home who have been with us since this happened | family support, collaboration among family members |
| INTERVIEW 15 ENGLISH VERSION.docx | Spiritual leaders come by because my father was a church elder, the chairperson of a committee at the church, so the choir comes to visit him, sing at home. So, the emotional support is there. | support from church leaders |
| INTERVIEW 15 ENGLISH VERSION.docx | That specific question wasn’t asked. We were just told we would be discharged, but no one asked whether we could take care of the patient at home. Even on the first day at the EMD, they just told us we would go home. It was I who said that I couldn’t take care of the patient; I initiated it, saying I’m not capable of taking care of the patient. | not asked about home environment |
| INTERVIEW 15 ENGLISH VERSION.docx | the shock of having a patient, we rushed to the hospital, and we forgot the insurance, and we had never been treated there before. We had forgotten the insurance number, | forget to carry NHIF card |
| INTERVIEW 15 ENGLISH VERSION.docx | So, all the initial treatment costs were about four hundred thousand shillings, we had to pay cash. We started using the insurance on the second day, but all the treatments at the EMD, radiology tests, blood tests, we had to pay in cash |  |
| INTERVIEW 15 ENGLISH VERSION.docx | So, all the initial treatment costs were about four hundred thousand shillings, we had to pay cash. We started using the insurance on the second day, but all the treatments at the EMD, radiology tests, blood tests, we had to pay in cash | forget to carry NHIF card |
| INTERVIEW 15 ENGLISH VERSION.docx | Mhm, maybe this one that's already being implemented, this one about ensuring, I don't know, incorporating insurance into NIDA. I went to verify his insurance, and they explained something like that to me, that even if you're sick and forget the insurance, you can receive treatment using the NIDA number. Because if we hadn't had any money at all that day, what would we have done? |  |
| INTERVIEW 15 ENGLISH VERSION.docx | Mhm, maybe this one that's already being implemented, this one about ensuring, I don't know, incorporating insurance into NIDA. I went to verify his insurance, and they explained something like that to me, that even if you're sick and forget the insurance, you can receive treatment using the NIDA number. Because if we hadn't had any money at all that day, what would we have done? But importantly is to link ICT systems across hospitals so that personal information can be retrieved across hospitals by using health insurance or national identity card to prevent delays in accessing care and duplication of investigations | link NIDA and insurance |
| INTERVIEW 15 ENGLISH VERSION.docx | Ah, another thing is, before they tell you to go home with the patient, they should ask if you're comfortable taking care of the patient at home. They shouldn't just say you'll go home with the patient. Can I really take care of my father, take him home and have him pee with me? Because there's a cultural aspect there, how will it be? Since he can't walk, I'll have to hold him to the washroom. Is it really possible? Mhm, it's difficult, so I think they should assess us. Are the relatives ready? How have we prepared before they discharge us? | suggest to have discharge readiness assessment |
| INTERVIEW 15 ENGLISH VERSION.docx | Mhm, another thing, maybe when you're going for these tests, sometimes the doctors in charge of the tests can be rude. You arrive, and they tell you, "Lie here," and I'm alone, pushing the wheelchair. If you let go of the wheelchair, you know it slides. Because they're using force to try and get up, so you ask, "Please help me, even just to hold the wheelchair so I can help my patient move over there." They're on the phone, and it's like, "This is not my job. The patient is yours. Once they're lying here, I will do the investigation." They say that's up to you. So, those doctors doing the tests, especially radiology, not all of them—over in MRI, CT-Scan, and so on, it was fine | poor communication from healthcare providers |
| INTERVIEW 15 ENGLISH VERSION.docx | Culture, that's the hard part at first, because now you're telling me to start taking off my father's shirt, start taking off my father's vest—we're not used to that. (Laughter ensues.) We're not used to that, and then he takes it lightly, so he tells me to take off my father's shirt. | respecting culture, culture issues in caring |
| INTERVIEW 15 ENGLISH VERSION.docx | So, there was a nurse who was calm there in radiology, so I would go to her and say, "Please help me take off my father's shirt." She would take off his shirt and put on his vest, but the doctor didn't care about that. For example, taking him to the washroom—there was a male nurse I asked the first day before my mother and siblings arrived, so I asked that male nurse, "Please help me take my father to the washroom." He took him. Eeh, so some were considerate, especially in the private ward—they were considerate because it was private. | respecting culture, culture issues in caring |
| INTERVIEW 15 ENGLISH VERSION.docx | But even that, when you say the patient should be transferred, when we transfer them, is there any difference? Because I asked the doctor, you say when a patient gets a stroke, here in the hospital you might get care that might reverse the condition or something like that, but even for us, there was no difference; it was just the same. | no thrombolytic therapy, delay in receiving proper treatment |
| INTERVIEW 15 ENGLISH VERSION.docx | So, we hurried to catch that window they’re talking about, but now that window, it’s like they didn’t regard it. So, this talk that why the six hours, there’s this and that, when you arrive within six hours, nothing really happens; they neglect you. Even if you stay two days, it doesn’t make a difference. | no thrombolytic therapy, delay in receiving proper treatment |
| INTERVIEW 8 ENGLISH VERSION.docx | We take her out for exercises and to the nearby hospital for therapy, little by little | exercises at nearby hospital |
| INTERVIEW 8 ENGLISH VERSION.docx | There are challenges, like work. You can’t perform well at work because of the patient. | stroke affect work performance of caregiver |
| INTERVIEW 8 ENGLISH VERSION.docx | I haven’t faced any challenges with the hospital, but the challenge is not going to work, not earning money, and things come to a standstill | stop working to nurse my mother, not earning money |
| INTERVIEW 8 ENGLISH VERSION.docx | Since I came here, the staff have been very good. They receive us well. For example, one day we came here, and she couldn’t walk. A young man, a staff member, helped us by bringing a wheelchair and assisted us until we got here. So, there is no difficulty at all. | support from healthcare providers, satisfaction with services, dissatisfied with nursing care, memorable good experience |
| INTERVIEW 8 ENGLISH VERSION.docx | The cooperation is great. They follow up well, the doctors are good, they listen to the patient well, and they want to know the patient’s history thoroughly. So, I see everything is going well. | cooperation with healthcare providers |
| INTERVIEW 8 ENGLISH VERSION.docx | For example, when we came the first time, the doctor wanted to know when she started getting sick. We explained which hospital we took her to initially, and he told us to start doing tests here again. We did the tests, and then he understood where the problem was | good communication with healthcare providers, interactions with healthcare providers, family involvement, caregiver involvement |
| INTERVIEW 8 ENGLISH VERSION.docx | It was good because we all went in together to see the doctor, so I knew everything. | caregiver involvement, family involvement, feelings of involvement in care, good communication with healthcare providers |
| INTERVIEW 8 ENGLISH VERSION.docx | Ahhh… they provided the information while we were all there. They explained everything step by step. | Information given step by step, information sharing |
| INTERVIEW 8 ENGLISH VERSION.docx | They said the patient indeed had a stroke, but they couldn’t say it was just a stroke. They mentioned there was also a tumor in the head, that’s what they told us. | good communication with healthcare providers, told about the diagnosis, information sharing |
| INTERVIEW 8 ENGLISH VERSION.docx | I thought it was normal because I felt it was better for her to go home sometimes rather than stay in the hospital. We live far away. | readiness for discharge, patient residence influence discharge |
| INTERVIEW 8 ENGLISH VERSION.docx | Maybe if they could increase the number of doctors to reduce the long queues. | increase staffing |
| INTERVIEW 14 ENGLISH VERSION.docx | As for the exact type of stroke, we’re not sure | unaware of stroke type |
| INTERVIEW 14 ENGLISH VERSION.docx | Yesterday, she was sent to a regional hospital, when suddenly her blood pressure spiked. She received initial treatment there with medication to lower her blood pressure. Then, she flew here this morning, and upon arrival, she underwent several tests—about five or six of them. Today was the day to receive the results. The findings show that she hasn’t suffered major effects other than what we can observe: paralysis on the right side, difficulty walking, and trouble eating. Her speech is also slightly affected.” | stroke onset, unaware of stroke signs, right sided stroke, failure to walk after stroke, trouble eating, loss of speech after stroke |
| INTERVIEW 14 ENGLISH VERSION.docx | We know she’ll need physical therapy, so we’ll follow their guidance.” | attend physiotherapy |
| INTERVIEW 14 ENGLISH VERSION.docx | “We haven’t made any specific requests. We believe things are going well.” | good services |
| INTERVIEW 14 ENGLISH VERSION.docx | The education was good. She also has diabetes. I remember she was on a different medication, but we asked about her blood sugar levels, which were slow to decrease. They adjusted her medication and explained that it would benefit her heart as well. Overall, we’re satisfied with the care. | good services, satisfaction with services, benefits of inquiry, support from healthcare providers, good communication with healthcare providers, asking questions to HCPs, medication adjustment made |
| INTERVIEW 14 ENGLISH VERSION.docx | However, one area for improvement is the network connectivity. We noticed interruptions, especially in communication.” | problem with network connection at the hospital |
| INTERVIEW 14 ENGLISH VERSION.docx | “They informed us in advance that she’d be discharged the next day. However, on the actual discharge day, there seemed to be some delay. We waited quite a while.” | discharge notice not given in advance |
| INTERVIEW 14 ENGLISH VERSION.docx | They had given us early notice, but when it came time to leave, there was a significant delay. | delays in discharge logistics |
| INTERVIEW 14 ENGLISH VERSION.docx | they told us she’d be discharged the next day, and we expected everything to be ready. However, it didn’t happen as smoothly | discharge notice not given in advance, delays in discharge logistics |
| INTERVIEW 14 ENGLISH VERSION.docx | They started her on insulin, but they hadn’t taught us how to administer it. So, on the day of discharge, I reminded the nurse that they had promised to teach us. She provided clear instructions on when and how to administer insulin, including demonstrations. We appreciated that. | taught to administer insulin injections, support from healthcare providers, good communication with healthcare providers, interactions with healthcare providers, asking questions to HCPs |
| INTERVIEW 14 ENGLISH VERSION.docx | They informed us about follow-up visits. They said to come back after a month, but if any issues arose, we should return earlier.” | encourage survivors to follow instructions, follow up date given |
| INTERVIEW 14 ENGLISH VERSION.docx | I’d suggest a more systematic approach. For instance, when we took another patient to abroad, they had a system where specialists would provide education. I expected a similar approach here. For example, a diabetes specialist could explain the condition, lifestyle changes, and the importance of adhering to treatment though radio or TV. | suggest need of specialist education |
| INTERVIEW 14 ENGLISH VERSION.docx | Also, printed materials would help—something that patients can refer to. Sometimes, relying solely on verbal instructions can be challenging, especially for older patients. Having written guidelines would be beneficial.” | suggest for printed stroke reference materials |
| INTERVIEW 12 ENGLISH VERSION.docx | We saw it was not normal, so we decided to take him somewhere. My younger brother took him to a hospital before reaching Tertiary hospital. What is the name of that hospital before Tertiary hospital? The regional one, I think. They went there, and he was referred to Tertiary hospital. | referred to big hospital |
| INTERVIEW 12 ENGLISH VERSION.docx | Yes, we stayed there for a few days. My younger brother was with him at Tertiary hospital. He was in a bad condition, so they transferred him by ambulance to here. | bad condition after stroke, used ambulance at national hospital |
| INTERVIEW 12 ENGLISH VERSION.docx | Here at the hospital. He was brought here to Tertiary hospital. | referred to national hospital |
| INTERVIEW 12 ENGLISH VERSION.docx | Through tests he was tested, and it was found that he had something in his head, blood clots or something. | informed of test results |
| INTERVIEW 12 ENGLISH VERSION.docx | Ah, my younger brother and other relatives handled that. My sisters and older brothers were in charge. | family involvement |
| INTERVIEW 12 ENGLISH VERSION.docx | In the ward… yes, I was there, bringing food | family involvement |
| INTERVIEW 12 ENGLISH VERSION.docx | Well… since I wasn’t always at the hospital, my younger brother was there | collaboration among family members |
| INTERVIEW 12 ENGLISH VERSION.docx | He said that our father was in the ward, and he had tubes, and until we brought him home, he was being fed through a nasal tube. We would feed him porridge or broth through the tube until he got better. So, until he was discharged, he was in that condition because I came and saw him. | patient change condition during clinic visit, feeding by relatives |
| INTERVIEW 12 ENGLISH VERSION.docx | In the ward… they were trying their best. From what I saw, they were attentive, taking care of the patients, setting up drips quickly. The ward had patients who were not fully conscious, so the staff were moving quickly and doing their best. | appreciations to healthcare providers |
| INTERVIEW 12 ENGLISH VERSION.docx | Ah, these illnesses require money. That’s the challenge. Sometimes the patient needs exercises, but at home, there was a doctor who used to come and do exercises with him. He charged ten thousand shillings per day. We went with him initially, but eventually, it became difficult to afford. So, the challenge is money because even for food, we struggle. My younger brother and I are the ones fighting to provide. | stroke care is expensive, exercise at home, exercise by private trainer, exercise by private trainer is costful, stopped exercise due to cost, financial struggle after stroke, collaboration among family members |
| INTERVIEW 12 ENGLISH VERSION.docx | They would ask if the patient had any issues, if we were doing exercises with him, and we would tell them we were. | interactions with healthcare providers, good communication with healthcare providers |
| INTERVIEW 12 ENGLISH VERSION.docx | As a family, we would sit and discuss, and if something was beyond our capability, we would go with what we could manage. | collaboration among family members, family support |
| INTERVIEW 12 ENGLISH VERSION.docx | for exercises, the patient needs to do exercises, take medication, and we should focus on certain foods, avoid fatty foods. We were advised to give him fish and avoid starchy foods. | diet instructions after discharge, medication instructions after discharge, exercise at home |
| INTERVIEW 12 ENGLISH VERSION.docx | Even I don’t fully understand… ; fatty foods are not good for someone with this condition. | inadequate diet education given |
| INTERVIEW 12 ENGLISH VERSION.docx | I think some family members were involved. | family involvement |
| INTERVIEW 12 ENGLISH VERSION.docx | They really tried their best because he was in a bad condition, and they did their best. | support from healthcare providers, appreciations to healthcare providers |
| INTERVIEW 12 ENGLISH VERSION.docx | I think healthcare providers should come to our communities and give us education about stroke. This can also help us to get aware about stroke and do checkup on time. For example I didn’t know he had stroke, I thought it was just a fever or other health issues. | unaware of stroke signs, screening for stroke, community campaigns for stroke |
| INTERVIEW 12 ENGLISH VERSION.docx | I would suggest improvement of stroke care services in regional hospitals because there are many patients in tertiary hospitals, services become too slow, and many people decide not to come. Other people wait until they are tired; you wait until you are tired, and patients sit until they are exhausted. So, the issue is time, time is the challenge. | waiting is long, stroke affect many people, improve primary care |
| INTERVIEW 12 ENGLISH VERSION.docx | I would ask those in charge of clinics to try to help people get services faster because you can come in the morning and leave in the afternoon. Meanwhile, the patient is hungry, and you yourself start feeling hungry. So, time is really the challenge. | waiting is long, suggest services to be faster |
| INTERVIEW 12 ENGLISH VERSION.docx | Maybe they should educate us on how to care for the patient because some of us don’t understand. For example, I noticed my father’s hand was swollen, and I asked the nurse why it was swollen. They said we should massage it. So, they should try to educate us because many of us don’t know. This is the first time I’m dealing with a stroke patient, so education would help. | Suggest to educate family members |
| INTERVIEW 12 ENGLISH VERSION.docx | Yes, even the types of food should be planned for this condition. They tell us to do exercises because food also contributes. | inadequate diet education given, improve diet education |
| INTERVIEW 12 ENGLISH VERSION.docx | I would suggest that stroke services should be expanded in primary care, because the waiting time in tertiary hospitals is really tiring. | Suggest expanding stroke care in primary care |
| INTERVIEW 12 ENGLISH VERSION.docx | They need three main things: proper medication, exercises, and a good diet. Those are the key things for this condition. | proper medication is key, exercise is key, good diet is key |
| INTERVIEW 12 ENGLISH VERSION.docx | But time is really a challenge. Time is a big issue for me, and I think for many others too. | waiting is long |
| INTERVIEW 12 ENGLISH VERSION.docx | You wait until you are tired. You expect to see the doctor, get a prescription, and leave, but the reality is different; that’s it. | waiting is long |
