# Supplementary material for: Perspectives of stroke survivors, caregivers and healthcare providers on improving access to stroke care services in Tanzania: A qualitative study
Source: PLoS One. 2026 Aug 3;21(8):e0328334. doi: 10.1371/journal.pone.0328334 (PMC13432098; doi:10.1371/journal.pone.0328334)
Supplement: Attachment — Submitted filename: Response to reviewers.pdf [file pone.0328334.s003.pdf]

Reviewer #1

The authors address a meaningful topic, and the inclusion of multiple stakeholder groups adds value to the discussion on stroke care access. They are commended for bringing these perspectives together in their work.

Table 1: Authors' responses to reviewer 1

	Comment	Response
	Abstract	
	The abstract summarises the study but does not clearly articulate the specific research gap driving the work. It reiterates broad LMIC challenges without explaining why a Tanzanian case-study is needed.	In Tanzania, delayed hospital presentation, weak referral systems, high out-of-pocket costs, shortages of stroke-ready facilities, and limited rehabilitation services contribute to preventable deaths and long-term disability. Although previous studies have described barriers to accessing stroke care services, few have explored practical strategies for improving access using the combined perspectives of stroke survivors, caregivers, and healthcare providers across the full stroke care pathway. This study explored strategies to improve equitable access to stroke care services in Tanzania by harnessing the perspectives of stroke survivors, caregivers, and healthcare providers across the stroke care continuum.
	The reference to international organisations does not strengthen the rationale and feels disconnected from the aim.	Well noted, the sentence has been removed.
	The methods contain unnecessary detail (e.g., software, ethics reference).	Well noted, the sentences have been removed.
	The results section presents broad system-level recommendations aligned with WHO building blocks, but offers little sense of participants' lived experiences.	Well, the current study focuses on strategies to improve equitable access to stroke care services and not the participants' lived experiences in accessing stroke care services.
	The conclusion introduces ideas (e.g., telestroke) not clearly grounded in the abstract's results.	The conclusion has been revised to clearly stress on the strategies to improve access to stroke cares services.

	Introduction	
	The rationale for the study is generic and unclear. The introduction summarises common LMIC stroke-care issues (LMIC burden, pre-hospital delays, limited imaging) but does not really clarify what this study adds beyond existing Tanzanian or East African qualitative work.	Thank you very much, we have revised this paragraph, and state that this study explores strategies for improving equitable access to stroke care services in Tanzania through the integrated perspectives of stroke survivors, caregivers, and healthcare providers. This qualitative inquiry seeks to generate evidence that can inform policy reforms, strengthen service delivery, and improve patient-centered stroke care delivery in LMICs
	Although the introduction outlines several well-known challenges within the Tanzanian stroke-care system, it does not yet articulate the specific gap this study intends to address. Clarifying what remains unknown, despite existing policy and system-level descriptions, would strengthen the rationale for the qualitative inquiry. Gaps might include: absence of Tanzanian studies integrating survivor, caregiver, and provider perspectives across the stroke pathway; limited work analysing solutions rather than only barrier; and limited use of health-system frameworks to structure qualitative findings. Without this, the study risks appearing descriptive.	Thank you very much, we have revised this paragraph, and state that; although previous studies in Tanzania have described barriers to accessing stroke care services, much of the existing literature focuses primarily on healthcare provider perspectives, or quantitative descriptions of service gaps. Limited qualitative research has simultaneously explored the experiences of stroke survivors, caregivers, and healthcare providers in accessing services across the stroke care pathway. Furthermore, most existing studies emphasize on barriers rather than identifying practical, context-relevant strategies for improving care access
	The extended list of international associations (pg 3-4 lines 17-11) somewhat shifts attention away from the local problem. Briefly acknowledging them is helpful, but the section would be stronger if more directly linked to why a locally grounded qualitative study in Tanzania is needed.	Thank you, we have urged that translating these global recommendations into practice may require understanding the local realities such as barriers that influence access to care, and opportunities for improvement.
	There is a lack of epidemiological grounding. No stroke services access metrics for Tanzania/LMICs are presented, leaving readers without a sense of why the access problem matters.	Well, we have considered epidemiological grounds in paragraph 1, and access metrics in paragraph 2-3.

	Methods	
	The statement that the clinic sees "90 stroke survivors daily" (pg 6 line 15) appears unusually high and implies roughly 450 stroke visits per week. This figure may need clarification or a bit more context.	The hospital serves as one of the country's largest stroke centers, and receives a high volume of stroke patients through both inpatient and outpatient services, with the neurology clinic managing approximately 90 patient visits per day. In addition, the hospital receives referrals from diverse regions across Tanzania.
	The reliance on WHO building blocks narrows analytic possibilities. These blocks are organisational categories, not explanatory theories. They are useful as descriptive heuristics but are not designed to capture relational aspects of care such as power dynamics, cultural beliefs, workflow dynamics, or mechanisms of access. Using them deductively may have funnelled findings into predictable categories and limited emergence of deeper insights. There are plenty of alternative frameworks (e.g., Levesque's Access to Care Framework, Penchansky & Thomas' Access Framework, etc.) that might have offered more explanatory value.	Thank you so much for enlightening us with healthcare access frameworks. But our aims to describe practical strategies to improving access to stroke care services. So the WHO framework is the most appropriate because it directly focuses on the health system as an implementable structure for change or improvement. It examines six actionable system domains which align closely with our study's objective of generating practical, individual to system-level strategies to improve access to stroke care services. This makes it particularly useful for translating qualitative findings into policy and health service improvements, especially in LMIC contexts like Tanzania where stroke care gaps are largely system-driven. In contrast, Levesque's Access to Care Framework is more patient-centred framework that focuses on dimensions of access, with less attention on structured or organized health system improvement interventions. Similarly, the Penchansky & Thomas framework is valuable for describing access gaps, but it does not explicitly map solutions to health system improvement.
	Study design & Sampling	
	The case-study justification is not well supported (pg 6 line 9). The manuscript states that case studies allow contextual	Thank you very much, we have reconsidered the study design. This study employed an descriptive qualitative design. This design

	understanding but does not define:<br>- what the "case" is (the hospital? the national system?).<br>- why MNH-Mloganzila is an appropriate case.<br>- the boundaries and scope of the case.<br>- how a single tertiary hospital can inform national-level access debates.<br>The way its written, "case study" functions as a label rather than a justified design.	allowed for the flexible and open-ended exploration of practical and context-specific strategies for improving equitable access to stroke services across the continuum of care in Tanzania.
	How did the authors monitor for saturation, and what is the justification for equal group sizes (pg 7 line 3).	Thank you very much. Equal group sizes were not predetermined as a strict requirement but were used as a practical guide to ensure balanced representation across the three stakeholder groups and allow comparison of perspectives.<br>Saturation was monitored concurrently during data collection and analysis through regular review of interview transcripts, coding patterns, and repetition of themes. Thematic saturation was achieved when no substantially new themes, insights, or variations were emerging across participant groups
	Restricting participation to those who can "communicate orally" (pg 7 line 7) excludes individuals with aphasia, severe disability, or altered consciousness, groups that face the greatest access barriers. This affects representativeness and should be acknowledged more explicitly.	Well, survivors with significant cognitive impairment, severe disability, or altered consciousness were excluded to ensure direct participation in in-depth interviews without proxy interpretation.
	Table 3 - Key professional cadres are missing (radiology, physiotherapy, OT, speech therapy, emergency medicine, EMS personnel, social workers) which undermines findings related to imaging, MDT function, rehabilitation gaps, and prehospital care. Only three medical doctors were included, yet many recommendations concern clinical decision pathways.	Although the primary participants included were nurses and medical doctors due to their central roles in day-to-day stroke management and continuity of care, other important multidisciplinary cadres such as physiotherapists, occupational therapists, speech therapists, radiology staff, emergency medicine personnel, and social workers were not included because of limited availability during the study period
	Data collection and analysis	

	Multiple interviewers with different training backgrounds (pg 7-8) but no detail on standardisation across the three.	Thank you very much. To ensure consistency and standardization across all interviews, all interviewers underwent prior training on the study objectives, interview guide, qualitative interviewing techniques, ethical considerations, probing strategies, and reflexivity to minimize interviewer bias. Mock interviews and regular debriefing sessions were also conducted to promote consistency in questioning style, probing depth, and interpretation of participant responses. Interviews were guided by the same semi-structured interview guide, allowing flexibility for follow-up questions
	Also, could the Interview context (hospital setting) have influenced provider responses?	Sure, the interview context may introduce bias. To reduce potential social desirability bias, participants were reassured that participation was voluntary, responses would remain confidential, no identifying information would be linked to their statements, and the study was intended for service improvement rather than performance evaluation
	Analytic approach contains contradictions - the authors state deductive analysis but also describe coding “across the dataset” (pg 8 line 13), which is inductive phrasing. Please clarify. Deductive use of WHO blocks likely contributed to predictable, surface-level themes and missed opportunities to explore richer, contradictory, or unexpected narratives.	Deductive coding using the WHO six health system building blocks framework provided an initial analytic structure. An inductive approach was then incorporated to allow identification of unexpected, contradictory, or context-specific issues that emerged beyond the predefined WHO domains. This combined deductive–inductive approach ensured that the analysis remained both theoretically informed and grounded in participants’ perspectives.
	Coding appears to have been conducted by one researcher. There is no mention of double coding, or intercoder dialogue, which may weaken the claims of trustworthiness.	Sure, a single-coder approach was used. To strengthen trustworthiness and reduce individual interpretive bias, all transcripts and coding decisions were independently reviewed by another member of the research team, followed by regular intercoder discussions to compare interpretations, resolve discrepancies, and refine the coding framework.
	Results	

	The themes closely mirror the WHO building blocks (pg 12-22), creating an impression of predetermined findings rather than emergent.	Thank you for this important observation. We agree that the close alignment of themes with the WHO health system building blocks may create the impression of predetermined findings. To address this, we have clarified in the methods section that the WHO framework was used as an initial sensitizing guide rather than a rigid coding template. Our analysis followed a flexible deductive–inductive approach, where the WHO building blocks helped to structure the inquiry around key health system domains, while still allowing unexpected, contradictory, and context-specific issues to emerge directly from participant narratives. Coding was not restricted to predefined categories, and themes were developed based on conceptual meaning and lived experiences rather than strict framework alignment. Some final themes naturally reflected WHO domains because participants frequently described solutions related to financing, workforce, service delivery, and governance. We have revised the manuscript to make this analytic process clearer and to emphasize that the findings were grounded in participant perspectives rather than imposed by the health system framework.
	While the study aims to capture participant perspectives, many of the results take the form of broad aspirational system improvements (e.g., calls for more trained EMS personnel, stroke units, or increased government financing) rather than grounded accounts of lived experience. This makes it difficult to distinguish whether the findings reflect participants' actual experiences navigating the system or their general policy preferences, and may dilute the experiential depth expected in qualitative work.	Thank you for this important observation. We agree that our findings appear more like broad policy recommendations than grounded lived experiences. These recommendations emerged from participants' direct experiences of navigating delays, financial hardship, referral challenges, and limited rehabilitation access across the stroke care pathway. The current study didn't aim to explore the participants lived experiences or challenges encountered in accessing stroke care services, because these challenges are well known in literature. However, the current study

		investigated possible strategies to access to stroke care services
	Several interesting contradictions in the data are not explored.<br>- Caregivers prefer direct doctor-led communication because nurse-delivered information feels incomplete (pg 16 lines 12-14), yet nurses are simultaneously positioned as key educators within the MDT (pg 15 lines 6-8).	Thank you. Caregivers expressed a clear preference for receiving stroke-related information directly from doctors rather than nurses, largely because physicians were perceived as having deeper clinical expertise and authority in diagnosis, prognosis, and treatment decisions. In contrast, nurse-led communication, although frequent and more accessible, was sometimes viewed as incomplete or limited in addressing complex medical questions, which led caregivers to seek further clarification from doctors whenever possible. This dynamic suggests that communication effectiveness in stroke care is influenced not only by the content of information provided but also by who delivers it, underscoring the need to strengthen trust in nurse-led education while promoting more structured, collaborative communication between doctors, nurses, and caregivers.
	- Caregivers call for structured discharge planning (pg 13 lines 17-22), while providers say this already happens, though inconsistently (pg 14 lines 1-3). Such tensions are analytically rich and could strengthen explanatory depth, but the rigid categorization caused by the chosen framework appears to have limited the authors' ability to explore them fully.	Thank you. The contrasting accounts between caregivers and healthcare providers regarding discharge planning present an important analytical tension within the findings. Caregivers describe discharge processes as unstructured, rushed, and lacking clear guidance on post-discharge care, rehabilitation, and warning signs, often leaving them feeling unprepared to manage stroke survivors at home. In contrast, healthcare providers indicate that discharge planning is routinely undertaken, though they acknowledged that its implementation is inconsistent due to workload pressures, staffing constraints, and limited time for detailed counselling. This divergence is analytically significant as it

		highlights a gap between intended clinical processes and patient-perceived realities of care delivery. Rather than indicating contradiction alone, these differing perspectives reveal systemic variability in how discharge planning is operationalised across settings hence requiring systemic improvement
	Structural determinants of access (transport, cost, rurality, gender, socio-economic factors, belief systems, traditional healers) receive minimal attention despite being central to access pathways.	These structural determinants were not explored in depth because the study's primary aim was to identify practical, health system–level strategies to improve access to stroke care rather than comprehensively analyse all upstream social determinants of health. The focus was therefore on actionable areas within the health system such as referral processes, workforce capacity, and service integration where feasible interventions could be developed. While participants did mention broader contextual barriers, these were not developed into standalone themes as they fall outside the study's strategy-oriented scope and would require a separate, more in-depth socio-cultural investigation
	Discussion	
	The tone resembles a policy report. It is dominated by high-income country examples (tele-stroke systems, mobile stroke units, wearable devices) (pg 22-26), which overshadows the Tanzanian context and do not meaningfully respond to the local health-system realities.	Thank you very much for this observation, the discussion has been revised to incorporate the Tanzanian context to meaningfully respond to the local health-system realities.
	The discussion scarcely engages with Tanzania. There is no reference to NHIF/CHF schemes, imaging capacity, workforce distribution, or national NCD policy. As written, the discussion could apply to almost any LMIC.	Thank you very much for this observation, discussion has been revised to incorporate the reference to national NCD policy and NHIF/CHF coverage
	External studies are summarised without clear integration back into the authors' own findings. This leaves the	Thank you very much for this observation, the external studies are now summarized with clear integration back into our findings

	reader uncertain how the study advances current knowledge.	
	Telemedicine is promoted as a “game changer” (pg 25 line 1), without acknowledging infrastructure constraints in Tanzania such as connectivity, electricity reliability, digital literacy, clinician training, or cost barriers.	Thank you very much for this observation, we have now acknowledged infrastructure constraints such as connectivity, electricity reliability, digital literacy, clinician training, and cost of care as possible implementation barriers to telestroke adoption in Tanzania.
	Writing & presentation	
	Frequent repetition of the same issues causing manuscript to be long (EMS delays, imaging shortages, staffing constraints).	Thank you very much, the paragraphs have been rephrased and made short and clear to avoid repetitions.
	Some editing needed (e.g. pg 2 line 3 “with particularly in LMICs”; pg 2 lines 6-7 “Most of LMICs lack of”; pg 24 line 3 “Despite of”)	Thank you very much, the editing has been made as suggested
	Some table entries are unclear (e.g., “closed hardware” pg 10).	Thank you very much, table entries have been revised, closed hardware means stopped working at hardware
	The conclusion is generic and does not reflect the specific contextual insights by participants from Tanzania.	Thank you very much, the conclusion has been revised to reflect the specific contextual insights by participants from Tanzania.
	Overall	
	Overall, I believe this is a relevant and important piece of work that would benefit from further refinement.	Thank you very much for insightful and thought-provoking comments that has helped us to refine our manuscript

Reviewer #2

This is an informative, and primarily narrative article highlighting the importance of engagement with the healthcare providers, patients and patient's families/caregivers to help improve the outcomes of stroke affected patients in low- and middle-income countries such as Tanzania. While there seems to have been an effort to address the increasing incidence of non-communicable diseases in these countries, there are inherent difficulties in tackling this challenge depending on the country.

The authors have tried to highlight these difficulties in a multi-layered manner and have made

some suggestions to address these difficulties; while these suggestions have some limitations, they may represent a starting point from a global perspective in addressing stroke prevention.

Table 2: Authors' responses to reviewer 2

	Comment	Response
	The narrative nature of this article does raise the potential for recall bias as well as potentially not truly representing a broad cross section of the society (which the authors do acknowledge). It may be helpful to have included patients/healthcare providers from a local/zonal hospital system as well to provide a better understanding of the challenges in stroke prevention in Tanzania.	Recruiting healthcare providers from regional and district hospitals was challenging primarily because the study was conducted at Muhimbili National Hospital–Mloganzila, a tertiary referral and national stroke center where most specialized stroke services are concentrated. Many stroke patients from zonal, regional and district hospitals are referred to this facility for advanced management, making it the most practical setting for accessing stroke survivors, caregivers, and providers directly involved in specialized stroke care. In addition, healthcare providers at lower-level facilities often have heavy clinical workloads, limited staffing, and fewer dedicated neurology or stroke care personnel, which made scheduling interviews difficult during the study period. Some providers were also less available due to administrative constraints, limited institutional support for research participation, and the absence of formal stroke units in many district hospitals. Resource and time limitations further restricted travel to multiple sites across different regions. As a result, the study prioritized providers working at the tertiary referral level while acknowledging that perspectives from regional and district hospitals would have strengthened understanding of referral pathways and pre-hospital access challenges. This has been recognized as a limitation of the study.
