# Supplementary material for: Perspectives of stroke survivors, caregivers and healthcare providers on improving access to stroke care services in Tanzania: A qualitative study
Source: PLoS One. 2026 Aug 3;21(8):e0328334. doi: 10.1371/journal.pone.0328334 (PMC13432098; doi:10.1371/journal.pone.0328334)
Supplement: Attachment — Submitted filename: Response to Response to Reviewer.pdf [file pone.0328334.s004.pdf]

Response to Response to Reviewer #2

We sincerely thank the reviewers for the thoughtful and constructive comments. We have carefully considered all suggestions and revised the manuscript accordingly. Our point-by-point responses are provided below.

Comment 1:

Thank you for the explanation - acknowledging it certainly helps. It would be helpful to explicitly state in this scenario that the findings may also not represent patients who do not have access to tertiary care in this scenario.

Response:

We thank the reviewer for this important observation. We agree that participants recruited from a tertiary hospital may not fully represent the experiences and perspectives of stroke survivors who are unable to access tertiary-level services. We have revised the limitations section to explicitly acknowledge that our findings may not reflect the needs and experiences of patients who face substantial barriers to reaching tertiary care facilities. This limitation has now been highlighted to improve the interpretation and transferability of the findings.

Comment 2:

We did note that in the abstract it is mentioned that "none have explored practical strategies for improving access" - it may be reasonable to consider re-wording it to allow a more conservative tone, since there are references to prior qualitative work performed in Tanzania.

Response:

We appreciate the reviewer's suggestion and agree that the original wording may have overstated the novelty of the study. To better acknowledge existing qualitative research on stroke care in Tanzania, we have revised the statement in the abstract and introduction to adopt a more conservative and balanced tone. Revision made: Although previous qualitative studies in Tanzania have identified barriers to accessing stroke care services, there remains limited evidence on strategies for improving access across the continuum of stroke care.

Comment 3:

Also noted is the fact that while the authors have stated they have not received funding for this work, there is mention of the study being funded by National Natural Science Foundation of China, as well as MUHAS-Higher Education for Economic Transformation in Tanzania, World Bank Project. It would do well to highlight this in the funding section, even if the authors have not received the funding directly.

Response:

We thank the reviewer for highlighting this inconsistency. We agree that transparency regarding funding support is important. We have carefully reviewed the funding statement and revised it to accurately reflect the sources of financial support and the nature of the support received.
Revision made: This study was supported by the National Natural Science Foundation of China and the Muhimbili University of Health and Allied Sciences (MUHAS) Higher Education for Economic Transformation (HEET) Project funded by the World Bank. The funding bodies had no role in the design of the study, data collection, analysis, interpretation of findings, or preparation of the manuscript.

Comment 4:

Finally, some of the citations have been duplicated - please re-check some of the references to remove the duplications.

Response:

We thank the reviewer for identifying this issue. We have thoroughly reviewed the reference list and in-text citations and removed duplicated references where identified. We also carefully cross-checked all citations to ensure consistency, accuracy, and compliance with the journal's referencing style.

We sincerely appreciate the reviewer’s valuable comments, which have helped improve the clarity, transparency, and rigor of the manuscript.
