# Supplementary material for: Perspectives of stroke survivors, caregivers and healthcare providers on improving access to stroke care services in Tanzania: A qualitative study
Source: PLoS One. 2026 Aug 3;21(8):e0328334. doi: 10.1371/journal.pone.0328334 (PMC13432098; doi:10.1371/journal.pone.0328334)
Supplement: Peer review history

# Peer review: Decision Letter 0

**DOI:** 10.1371/journal.pone.0328334.r001

**Authors:** Shashank Shekhar

16 Apr 2026

PONE-D-25-34987

Perspectives of stroke survivors, caregivers and healthcare providers on improving access to stroke care services: a qualitative study

PLOS One

Dear Dr. Michael,

Thank you for submitting your manuscript to PLOS ONE. After careful consideration, we feel that it has merit but does not fully meet PLOS ONE’s publication criteria as it currently stands. Therefore, we invite you to submit a revised version of the manuscript that addresses the points raised during the review process.

Please submit your revised manuscript by May 31 2026 11:59PM. If you will need more time than this to complete your revisions, please reply to this message or contact the journal office at plosone@plos.org. When you're ready to submit your revision, log on to https://www.editorialmanager.com/pone/ and select the 'Submissions Needing Revision' folder to locate your manuscript file.

Please include the following items when submitting your revised manuscript:

- A letter that responds to each point raised by the academic editor and reviewer(s). You should upload this letter as a separate file labeled 'Response to Reviewers'.
- A marked-up copy of your manuscript that highlights changes made to the original version. You should upload this as a separate file labeled 'Revised Manuscript with Track Changes'.
- An unmarked version of your revised paper without tracked changes. You should upload this as a separate file labeled 'Manuscript'.

If you would like to make changes to your financial disclosure, please include your updated statement in your cover letter. Guidelines for resubmitting your figure files are available below the reviewer comments at the end of this letter.

If applicable, we recommend that you deposit your laboratory protocols in protocols.io to enhance the reproducibility of your results. Protocols.io assigns your protocol its own identifier (DOI) so that it can be cited independently in the future. For instructions see: https://journals.plos.org/plosone/s/submission-guidelines#loc-laboratory-protocols. Additionally, PLOS ONE offers an option for publishing peer-reviewed Lab Protocol articles, which describe protocols hosted on protocols.io. Read more information on sharing protocols at https://plos.org/protocols?utm_medium=editorial-email&utm_source=authorletters&utm_campaign=protocols.

As the corresponding author, your ORCID iD is verified in the submission system and will appear in the published article. PLOS supports the use of ORCID, and we encourage all coauthors to register for an ORCID iD and use it as well. Please encourage your coauthors to verify their ORCID iD within the submission system before final acceptance, as unverified ORCID iDs will not appear in the published article. *Only*  the individual author can complete the verification step; PLOS staff *cannot*  verify ORCID iDs on behalf of authors.

We look forward to receiving your revised manuscript.

Kind regards,

Shashank Shekhar, MD

Academic Editor

PLOS One

Journal Requirements:

When submitting your revision, we need you to address these additional requirements.

1.Please ensure that your manuscript meets PLOS ONE's style requirements, including those for file naming. The PLOS ONE style templates can be found at

https://journals.plos.org/plosone/s/file?id=wjVg/PLOSOne_formatting_sample_main_body.pdf and

https://journals.plos.org/plosone/s/file?id=ba62/PLOSOne_formatting_sample_title_authors_affiliations.pdf

2. We note that your Data Availability Statement is currently as follows: All relevant data are within the manuscript and in Supporting Information files.

Please confirm at this time whether or not your submission contains all raw data required to replicate the results of your study. Authors must share the “minimal data set” for their submission. PLOS defines the minimal data set to consist of the data required to replicate all study findings reported in the article, as well as related metadata and methods (https://journals.plos.org/plosone/s/data-availability#loc-minimal-data-set-definition).

For example, authors should submit the following data:

- The values behind the means, standard deviations and other measures reported;

- The values used to build graphs;

- The points extracted from images for analysis.

Authors do not need to submit their entire data set if only a portion of the data was used in the reported study.

If your submission does not contain these data, please either upload them as Supporting Information files or deposit them to a stable, public repository and provide us with the relevant URLs, DOIs, or accession numbers. For a list of recommended repositories, please see https://journals.plos.org/plosone/s/recommended-repositories.

If there are ethical or legal restrictions on sharing a de-identified data set, please explain them in detail (e.g., data contain potentially sensitive information, data are owned by a third-party organization, etc.) and who has imposed them (e.g., an ethics committee). Please also provide contact information for a data access committee, ethics committee, or other institutional body to which data requests may be sent. If data are owned by a third party, please indicate how others may request data access.

3. Please include a caption for figure 1.

4. Please include captions for your Supporting Information files at the end of your manuscript, and update any in-text citations to match accordingly. Please see our Supporting Information guidelines for more information: http://journals.plos.org/plosone/s/supporting-information.

5. If the reviewer comments include a recommendation to cite specific previously published works, please review and evaluate these publications to determine whether they are relevant and should be cited. There is no requirement to cite these works unless the editor has indicated otherwise.

Additional Editor Comments:

There are some meaningful feedback and comments suggested by the reviewer.

[Note: HTML markup is below. Please do not edit.]

Reviewers' comments:

Reviewer's Responses to Questions

**Comments to the Author**

1. Is the manuscript technically sound, and do the data support the conclusions?

The manuscript must describe a technically sound piece of scientific research with data that supports the conclusions. Experiments must have been conducted rigorously, with appropriate controls, replication, and sample sizes. The conclusions must be drawn appropriately based on the data presented.

Reviewer #1: Partly

Reviewer #2: Yes

**********

2. Has the statistical analysis been performed appropriately and rigorously?

Reviewer #1: N/A

Reviewer #2: I Don't Know

**********

3. Have the authors made all data underlying the findings in their manuscript fully available?

The PLOS Data policy requires authors to make all data underlying the findings described in their manuscript fully available without restriction, with rare exception (please refer to the Data Availability Statement in the manuscript PDF file). The data should be provided as part of the manuscript or its supporting information, or deposited to a public repository. For example, in addition to summary statistics, the data points behind means, medians and variance measures should be available. If there are restrictions on publicly sharing data—e.g. participant privacy or use of data from a third party—those must be specified.

Reviewer #1: Yes

Reviewer #2: Yes

**********

4. Is the manuscript presented in an intelligible fashion and written in standard English?

PLOS ONE does not copyedit accepted manuscripts, so the language in submitted articles must be clear, correct, and unambiguous. Any typographical or grammatical errors should be corrected at revision, so please note any specific errors here.

Reviewer #1: Yes

Reviewer #2: Yes

**********

5. Review Comments to the Author

Please use the space provided to explain your answers to the questions above. You may also include additional comments for the author, including concerns about dual publication, research ethics, or publication ethics. (Please upload your review as an attachment if it exceeds 20,000 characters)

Reviewer #1: The authors address a meaningful topic, and the inclusion of multiple stakeholder groups adds value to the discussion on stroke care access. They are commended for bringing these perspectives together in their work.

Background

• The rationale for the study is generic and unclear. The introduction summarises common LMIC stroke-care issues (LMIC burden, pre-hospital delays, limited imaging) but does not really clarify what this study adds beyond existing Tanzanian or East African qualitative work.

• Although the introduction outlines several well-known challenges within the Tanzanian stroke-care system, it does not yet articulate the specific gap this study intends to address. Clarifying what remains unknown, despite existing policy and system-level descriptions, would strengthen the rationale for the qualitative inquiry.

Gaps might include:

- absence of Tanzanian studies integrating survivor, caregiver, and provider perspectives across the stroke pathway

- limited work analysing solutions rather than only barriers

- limited use of health-system frameworks to structure qualitative findings

Without this, the study risks appearing descriptive.

• The extended list of international associations (pg 3-4 lines 17-11) somewhat shifts attention away from the local problem. Briefly acknowledging them is helpful, but the section would be stronger if more directly linked to why a locally grounded qualitative study in Tanzania is needed.

• There is a lack of epidemiological grounding. No stroke services access metrics for Tanzania/LMICs are presented, leaving readers without a sense of why the access problem matters.

• The statement that the clinic sees “90 stroke survivors daily” (pg 6 line 15) appears unusually high and implies roughly 450 stroke visits per week. This figure may need clarification or a bit more context.

• The reliance on WHO building blocks narrows analytic possibilities. These blocks are organisational categories, not explanatory theories. They are useful as descriptive heuristics but are not designed to capture relational aspects of care such as power dynamics, cultural beliefs, workflow dynamics, or mechanisms of access. Using them deductively may have funnelled findings into predictable categories and limited emergence of deeper insights. There are plenty of alternative frameworks (e.g., Levesque’s Access to Care Framework, Penchansky & Thomas’ Access Framework, etc.) that might have offered more explanatory value.

Study design & Sampling

• The case-study justification is not well supported (pg 6 line 9). The manuscript states that case studies allow contextual understanding but does not define:

- what the “case” is (the hospital? the national system?).

- why MNH-Mloganzila is an appropriate case.

- the boundaries and scope of the case.

- how a single tertiary hospital can inform national-level access debates.

The way its written, “case study” functions as a label rather than a justified design.

• How did the authors monitor for saturation, and what is the justification for equal group sizes (pg 7 line 3).

• Restricting participation to those who can “communicate orally” (pg 7 line 7) excludes individuals with aphasia, severe disability, or altered consciousness, groups that face the greatest access barriers. This affects representativeness and should be acknowledged more explicitly.

• Table 3 - Key professional cadres are missing (radiology, physiotherapy, OT, speech therapy, emergency medicine, EMS personnel, social workers) which undermines findings related to imaging, MDT function, rehabilitation gaps, and prehospital care. Only three medical doctors were included, yet many recommendations concern clinical decision pathways.

Data collection and analysis

• Multiple interviewers with different training backgrounds (pg 7-8) but no detail on standardisation across the three. Also, could the Interview context (hospital setting) have influenced provider responses?

• Analytic approach contains contradictions - the authors state deductive analysis but also describe coding “across the dataset” (pg 8 line 13), which is inductive phrasing. Please clarify

• Coding appears to have been conducted by one researcher. There is no mention of double coding, or intercoder dialogue, which may weaken the claims of trustworthiness.

• Deductive use of WHO blocks likely contributed to predictable, surface-level themes and missed opportunities to explore richer, contradictory, or unexpected narratives.

Results

• The themes closely mirror the WHO building blocks (pg 12-22), creating an impression of predetermined findings rather than emergent.

• While the study aims to capture participant perspectives, many of the results take the form of broad aspirational system improvements (e.g., calls for more trained EMS personnel, stroke units, or increased government financing) rather than grounded accounts of lived experience. This makes it difficult to distinguish whether the findings reflect participants’ actual experiences navigating the system or their general policy preferences, and may dilute the experiential depth expected in qualitative work.

• Several interesting contradictions in the data are not explored.

- Caregivers prefer direct doctor-led communication because nurse-delivered information feels incomplete (pg 16 lines 12-14), yet nurses are simultaneously positioned as key educators within the MDT (pg 15 lines 6-8).

- Caregivers call for structured discharge planning (pg 13 lines 17-22), while providers say this already happens, though inconsistently (pg 14 lines 1-3).

Such tensions are analytically rich and could strengthen explanatory depth, but the rigid categorization caused by the chosen framework appears to have limited the authors’ ability to explore them fully.

• Structural determinants of access (transport, cost, rurality, gender, socio-economic factors, belief systems, traditional healers) receive minimal attention despite being central to access pathways.

Discussion

• The tone resembles a policy report. It is dominated by high-income country examples (tele-stroke systems, mobile stroke units, wearable devices) (pg 22-26), which overshadows the Tanzanian context and do not meaningfully respond to the local health-system realities.

• The discussion scarcely engages with Tanzania. There is no reference to NHIF/CHF schemes, imaging capacity, workforce distribution, or national NCD policy. As written, the discussion could apply to almost any LMIC.

• External studies are summarised without clear integration back into the authors’ own findings. This leaves the reader uncertain how the study advances current knowledge.

• Telemedicine is promoted as a “game changer” (pg 25 line 1), without acknowledging infrastructure constraints in Tanzania such as connectivity, electricity reliability, digital literacy, clinician training, or cost barriers.

Writing & presentation

• Frequent repetition of the same issues causing manuscript to be long (EMS delays, imaging shortages, staffing constraints).

• Some editing needed (e.g. pg 2 line 3 “with particularly in LMICs”; pg 2 lines 6-7 “Most of LMICs lack of”; pg 24 line 3 “Despite of”)

• Some table entries are unclear (e.g., “closed hardware” pg 10).

• The conclusion is generic and does not reflect the specific contextual insights by participants from Tanzania.

Abstract

• The abstract summarises the study but does not clearly articulate the specific research gap driving the work. It reiterates broad LMIC challenges without explaining why a Tanzanian case-study is needed.

• The reference to international organisations does not strengthen the rationale and feels disconnected from the aim.

• The methods contain unnecessary detail (e.g., software, ethics reference).

• The results section presents broad system-level recommendations aligned with WHO building blocks, but offers little sense of participants’ lived experiences.

• The conclusion introduces ideas (e.g., telestroke) not clearly grounded in the abstract’s results.

Overall, I believe this is a relevant and important piece of work that would benefit from further refinement.

Reviewer #2: This is an informative, and primarily narrative article highlighting the importance of engagement with the healthcare providers, patients and patient's families/caregivers to help improve the outcomes of stroke affected patients in low- and middle-income countries such as Tanzania. While there seems to have been an effort to address the increasing incidence of non-communicable diseases in these countries, there are inherent difficulties in tackling this challenge depending on the country.

The authors have tried to highlight these difficulties in a multi-layered manner and have made some suggestions to address these difficulties; while these suggestions have some limitations, they may represent a starting point from a global perspective in addressing stroke prevention.

The narrative nature of this article does raise the potential for recall bias as well as potentially not truly representing a broad cross section of the society (which the authors do acknowledge). It may be helpful to have included patients/healthcare providers from a local/zonal hospital system as well to provide a better understanding of the challenges in stroke prevention in Tanzania.

**********

6. PLOS authors have the option to publish the peer review history of their article (what does this mean?). If published, this will include your full peer review and any attached files.

If you choose “no”, your identity will remain anonymous but your review may still be made public.

**Do you want your identity to be public for this peer review?**  For information about this choice, including consent withdrawal, please see our Privacy Policy.

Reviewer #1: **Yes:**  Moyahabo J Rampya

Reviewer #2: No

**********

[NOTE: If reviewer comments were submitted as an attachment file, they will be attached to this email and accessible via the submission site. Please log into your account, locate the manuscript record, and check for the action link "View Attachments". If this link does not appear, there are no attachment files.]

To ensure your figures meet our technical requirements, please review our figure guidelines: https://journals.plos.org/plosone/s/figures

You may also use PLOS’s free figure tool, NAAS, to help you prepare publication quality figures: https://journals.plos.org/plosone/s/figures#loc-tools-for-figure-preparation.

NAAS will assess whether your figures meet our technical requirements by comparing each figure against our figure specifications.

# Author comment: Author response to Decision Letter 1

**DOI:** 10.1371/journal.pone.0328334.r002

7 May 2026

Thank you so much for the valuable comments and feedback.

We have gone through all raised issues in the manuscript, we have addressed all the comments as attached in the response to reviewer file.

1. We have revised the abstract to articulate the specific research gap driving our work and explaining why a Tanzanian case-study is needed. We have emphasized that our work focuses on identifying key strategies to improve access and not the access barriers.

2. We have used the WHO health system building block because it offers domains that can be explored and give rise rise to real-world solutions to accessing or improving stroke care in our setting.

3. The introduction has been revised to include the local context situations, and highlighting the rationale of this study. We have also considered epidemiological grounds from global, regional and national level.

4. The method section has also been revised, from the study design to read a descriptive qualitative study since it entails to describe participants perceived strategies to improve access to stroke care services.

5. Equal group sizes were not predetermined as a strict requirement but were used as a practical guide to ensure balanced representation across the three stakeholder groups and allow comparison of perspectives.

6. Although the primary participants included were nurses and medical doctors due to their central roles in day-to-day stroke management and continuity of care, other important multidisciplinary cadres such as physiotherapists, occupational therapists, speech therapists, radiology staff, emergency medicine personnel, and social workers were not included because of limited availability during the study period.

7. To ensure consistency and standardization across all interviews, all interviewers underwent prior training on the study objectives, interview guide, qualitative interviewing techniques, ethical considerations, probing strategies, and reflexivity to minimize interviewer bias. Mock interviews and regular debriefing sessions were also conducted to promote consistency in questioning style, probing depth, and interpretation of participant responses. Interviews were guided by the same semi-structured interview guide, allowing flexibility for follow-up questions.

8. To reduce potential social desirability bias, participants were reassured that participation was voluntary, responses would remain confidential, no identifying information would be linked to their statements, and the study was intended for service improvement rather than performance evaluation.

9. Deductive coding using the WHO six health system building blocks framework provided an initial analytic structure. An inductive approach was then incorporated to allow identification of unexpected, contradictory, or context-specific issues that emerged beyond the predefined WHO domains. This combined deductive–inductive approach ensured that the analysis remained both theoretically informed and grounded in participants’ perspectives.

10. To strengthen trustworthiness and reduce individual interpretive bias, all transcripts and coding decisions were independently reviewed by another member of the research team, followed by regular intercoder discussions to compare interpretations, resolve discrepancies, and refine the coding framework.

11. survivors with significant cognitive impairment, severe disability, or altered consciousness were excluded to ensure direct participation in in-depth interviews without proxy interpretation.

12. The result section has been revised, extra codes were added to enhance trustworthiness of the findings.

13. The discussion has been revised to incorporate the Tanzanian context to meaningfully respond to the local health-system realities.

14. the conclusion has been revised to reflect the specific contextual insights by participants from Tanzania.

15. Minor edits have been made.

2.

Attachment Submitted filename: Response to reviewers.pdf

# Peer review: Decision Letter 1

**DOI:** 10.1371/journal.pone.0328334.r003

**Authors:** Shashank Shekhar

1 Jun 2026

PONE-D-25-34987R1

Perspectives of stroke survivors, caregivers and healthcare providers on improving access to stroke care services: a qualitative study

PLOS One

Dear Dr. Michael,

Thank you for submitting your manuscript to PLOS ONE. After careful consideration, we feel that it has merit but does not fully meet PLOS ONE’s publication criteria as it currently stands. Therefore, we invite you to submit a revised version of the manuscript that addresses the points raised during the review process.

There is still a few minor points that require revision. Kindly go over point by point and please resubmit.

Please submit your revised manuscript by Jul 16 2026 11:59PM. If you will need more time than this to complete your revisions, please reply to this message or contact the journal office at plosone@plos.org. When you're ready to submit your revision, log on to https://www.editorialmanager.com/pone/ and select the 'Submissions Needing Revision' folder to locate your manuscript file.

Please include the following items when submitting your revised manuscript:

- A letter that responds to each point raised by the academic editor and reviewer(s). You should upload this letter as a separate file labeled 'Response to Reviewers'.
- A marked-up copy of your manuscript that highlights changes made to the original version. You should upload this as a separate file labeled 'Revised Manuscript with Track Changes'.
- An unmarked version of your revised paper without tracked changes. You should upload this as a separate file labeled 'Manuscript'.

If you would like to make changes to your financial disclosure, please include your updated statement in your cover letter. Guidelines for resubmitting your figure files are available below the reviewer comments at the end of this letter.

If applicable, we recommend that you deposit your laboratory protocols in protocols.io to enhance the reproducibility of your results. Protocols.io assigns your protocol its own identifier (DOI) so that it can be cited independently in the future. For instructions see: https://journals.plos.org/plosone/s/submission-guidelines#loc-laboratory-protocols. Additionally, PLOS ONE offers an option for publishing peer-reviewed Lab Protocol articles, which describe protocols hosted on protocols.io. Read more information on sharing protocols at https://plos.org/protocols?utm_medium=editorial-email&utm_source=authorletters&utm_campaign=protocols.

As the corresponding author, your ORCID iD is verified in the submission system and will appear in the published article. PLOS supports the use of ORCID, and we encourage all coauthors to register for an ORCID iD and use it as well. Please encourage your coauthors to verify their ORCID iD within the submission system before final acceptance, as unverified ORCID iDs will not appear in the published article. *Only*  the individual author can complete the verification step; PLOS staff *cannot*  verify ORCID iDs on behalf of authors.

We look forward to receiving your revised manuscript.

Kind regards,

Shashank Shekhar, MD

Academic Editor

PLOS One

Journal Requirements:

1. If the reviewer comments include a recommendation to cite specific previously published works, please review and evaluate these publications to determine whether they are relevant and should be cited. There is no requirement to cite these works unless the editor has indicated otherwise.

2. Please review your reference list to ensure that it is complete and correct. If you have cited papers that have been retracted, please include the rationale for doing so in the manuscript text, or remove these references and replace them with relevant current references. Any changes to the reference list should be mentioned in the rebuttal letter that accompanies your revised manuscript. If you need to cite a retracted article, indicate the article’s retracted status in the References list and also include a citation and full reference for the retraction notice.

[Note: HTML markup is below. Please do not edit.]

Reviewers' comments:

Reviewer's Responses to Questions

**Comments to the Author**

1. If the authors have adequately addressed your comments raised in a previous round of review and you feel that this manuscript is now acceptable for publication, you may indicate that here to bypass the “Comments to the Author” section, enter your conflict of interest statement in the “Confidential to Editor” section, and submit your "Accept" recommendation.

Reviewer #1: All comments have been addressed

Reviewer #2: All comments have been addressed

**********

2. Is the manuscript technically sound, and do the data support the conclusions?

The manuscript must describe a technically sound piece of scientific research with data that supports the conclusions. Experiments must have been conducted rigorously, with appropriate controls, replication, and sample sizes. The conclusions must be drawn appropriately based on the data presented.

Reviewer #1: Yes

Reviewer #2: Partly

**********

3. Has the statistical analysis been performed appropriately and rigorously?

Reviewer #1: N/A

Reviewer #2: I Don't Know

**********

4. Have the authors made all data underlying the findings in their manuscript fully available?

The PLOS Data policy requires authors to make all data underlying the findings described in their manuscript fully available without restriction, with rare exception (please refer to the Data Availability Statement in the manuscript PDF file). The data should be provided as part of the manuscript or its supporting information, or deposited to a public repository. For example, in addition to summary statistics, the data points behind means, medians and variance measures should be available. If there are restrictions on publicly sharing data—e.g. participant privacy or use of data from a third party—those must be specified.

Reviewer #1: Yes

Reviewer #2: Yes

**********

5. Is the manuscript presented in an intelligible fashion and written in standard English?

PLOS ONE does not copyedit accepted manuscripts, so the language in submitted articles must be clear, correct, and unambiguous. Any typographical or grammatical errors should be corrected at revision, so please note any specific errors here.

Reviewer #1: Yes

Reviewer #2: No

**********

6. Review Comments to the Author

Please use the space provided to explain your answers to the questions above. You may also include additional comments for the author, including concerns about dual publication, research ethics, or publication ethics. (Please upload your review as an attachment if it exceeds 20,000 characters)

Reviewer #1: (No Response)

Reviewer #2: Thank you for the explanation - acknowledging it certainly helps. It would be helpful to explicitly state in this scenario that the findings may also not represent patients who do not have access to tertiary care in this scenario.

We did note that in the abstract it is mentioned that "none have explored practical strategies for improving access" - it may be reasonable to consider re-wording it to allow a more conservative tone, since there are references to prior qualitative work performed in Tanzania.

Also noted is the fact that while the authors have stated they have not received funding for this work, there is mention of the study being funded by National Natural Science Foundation of China, as well as

MUHAS-Higher Education for Economic Transformation in Tanzania, World Bank Project. it would do well to highlight this in the funding section, even if the authors have not received the funding directly.

Finally, some of the citations have been duplicated - please re-check some of the references to remove the duplications.

**********

7. PLOS authors have the option to publish the peer review history of their article (what does this mean?). If published, this will include your full peer review and any attached files.

If you choose “no”, your identity will remain anonymous but your review may still be made public.

**Do you want your identity to be public for this peer review?**  For information about this choice, including consent withdrawal, please see our Privacy Policy.

Reviewer #1: **Yes:**  Moyahabo J. Rampya

Reviewer #2: No

**********

[NOTE: If reviewer comments were submitted as an attachment file, they will be attached to this email and accessible via the submission site. Please log into your account, locate the manuscript record, and check for the action link "View Attachments". If this link does not appear, there are no attachment files.]

To ensure your figures meet our technical requirements, please review our figure guidelines: https://journals.plos.org/plosone/s/figures

You may also use PLOS’s free figure tool, NAAS, to help you prepare publication quality figures: https://journals.plos.org/plosone/s/figures#loc-tools-for-figure-preparation.

NAAS will assess whether your figures meet our technical requirements by comparing each figure against our figure specifications.

# Author comment: Author response to Decision Letter 2

**DOI:** 10.1371/journal.pone.0328334.r004

2 Jun 2026

Response to Response to Reviewer #2

We sincerely thank the reviewers for the thoughtful and constructive comments. We have carefully considered all suggestions and revised the manuscript accordingly. Our point-by-point responses are provided below.

Comment 1:

Thank you for the explanation - acknowledging it certainly helps. It would be helpful to explicitly state in this scenario that the findings may also not represent patients who do not have access to tertiary care in this scenario.

Response:

We thank the reviewer for this important observation. We agree that participants recruited from a tertiary hospital may not fully represent the experiences and perspectives of stroke survivors who are unable to access tertiary-level services. We have revised the limitations section to explicitly acknowledge that our findings may not reflect the needs and experiences of patients who face substantial barriers to reaching tertiary care facilities. This limitation has now been highlighted to improve the interpretation and transferability of the findings.

Comment 2:

We did note that in the abstract it is mentioned that "none have explored practical strategies for improving access" - it may be reasonable to consider re-wording it to allow a more conservative tone, since there are references to prior qualitative work performed in Tanzania.

Response:

We appreciate the reviewer’s suggestion and agree that the original wording may have overstated the novelty of the study. To better acknowledge existing qualitative research on stroke care in Tanzania, we have revised the statement in the abstract and introduction to adopt a more conservative and balanced tone. Revision made: Although previous qualitative studies in Tanzania have identified barriers to accessing stroke care services, there remains limited evidence on strategies for improving access across the continuum of stroke care.

Comment 3:

Also noted is the fact that while the authors have stated they have not received funding for this work, there is mention of the study being funded by National Natural Science Foundation of China, as well as MUHAS-Higher Education for Economic Transformation in Tanzania, World Bank Project. It would do well to highlight this in the funding section, even if the authors have not received the funding directly.

Response:

We thank the reviewer for highlighting this inconsistency. We agree that transparency regarding funding support is important. We have carefully reviewed the funding statement and revised it to accurately reflect the sources of financial support and the nature of the support received. Revision made: This study was supported by the National Natural Science Foundation of China and the Muhimbili University of Health and Allied Sciences (MUHAS) Higher Education for Economic Transformation (HEET) Project funded by the World Bank. The funding bodies had no role in the design of the study, data collection, analysis, interpretation of findings, or preparation of the manuscript.

Comment 4:

Finally, some of the citations have been duplicated - please re-check some of the references to remove the duplications.

Response:

We thank the reviewer for identifying this issue. We have thoroughly reviewed the reference list and in-text citations and removed duplicated references where identified. We also carefully cross-checked all citations to ensure consistency, accuracy, and compliance with the journal's referencing style.

We sincerely appreciate the reviewer’s valuable comments, which have helped improve the clarity, transparency, and rigor of the manuscript.

Attachment Submitted filename: Response to Response to Reviewer.pdf

# Editor report: Decision Letter 2

**DOI:** 10.1371/journal.pone.0328334.r005

**Authors:** Shashank Shekhar

7 Jul 2026

Perspectives of stroke survivors, caregivers and healthcare providers on improving access to stroke care services in Tanzania: a qualitative study

PONE-D-25-34987R2

Dear Dr. Michael,

We’re pleased to inform you that your manuscript has been judged scientifically suitable for publication and will be formally accepted for publication once it meets all outstanding technical requirements.

Within one week, you’ll receive an e-mail detailing the required amendments. When these have been addressed, you’ll receive a formal acceptance letter and your manuscript will be scheduled for publication.

An invoice will be generated when your article is formally accepted. Please note, if your institution has a publishing partnership with PLOS and your article meets the relevant criteria, all or part of your publication costs will be covered. Please make sure your user information is up-to-date by logging into Editorial Manager at Editorial Manager® and clicking the ‘Update My Information' link at the top of the page. For questions related to billing, please contact billing support.

If your institution or institutions have a press office, please notify them about your upcoming paper to help maximize its impact. If they’ll be preparing press materials, please inform our press team as soon as possible -- no later than 48 hours after receiving the formal acceptance. Your manuscript will remain under strict press embargo until 2 pm Eastern Time on the date of publication. For more information, please contact onepress@plos.org.

Kind regards,

Shashank Shekhar, MD

Academic Editor

PLOS One

Additional Editor Comments (optional):

Reviewers' comments:

# Editor report: Acceptance letter

**DOI:** 10.1371/journal.pone.0328334.r006

**Authors:** Shashank Shekhar

PONE-D-25-34987R2

PLOS One

Dear Dr. Michael,

I'm pleased to inform you that your manuscript has been deemed suitable for publication in PLOS One. Congratulations! Your manuscript is now being handed over to our production team.

At this stage, our production department will prepare your paper for publication. This includes ensuring the following:

* All references, tables, and figures are properly cited

* All relevant supporting information is included in the manuscript submission,

* There are no issues that prevent the paper from being properly typeset

You will receive further instructions from the production team, including instructions on how to review your proof when it is ready. Please keep in mind that we are working through a large volume of accepted articles, so please give us a few days to review your paper and let you know the next and final steps.

Lastly, if your institution or institutions have a press office, please let them know about your upcoming paper now to help maximize its impact. If they'll be preparing press materials, please inform our press team within the next 48 hours. Your manuscript will remain under strict press embargo until 2 pm Eastern Time on the date of publication. For more information, please contact onepress@plos.org.

You will receive an invoice from PLOS for your publication fee after your manuscript has reached the completed accept phase. If you receive an email requesting payment before acceptance or for any other service, this may be a phishing scheme. Learn how to identify phishing emails and protect your accounts at https://explore.plos.org/phishing.

If we can help with anything else, please email us at customercare@plos.org.

Thank you for submitting your work to PLOS ONE and supporting open access.

Kind regards,

PLOS ONE Editorial Office Staff

on behalf of

Dr. Shashank Shekhar

Academic Editor

PLOS One
